# Transgenic mouse model of IgM^+^ lymphoproliferative disease mimicking Waldenström macroglobulinemia

**DOI:** 10.1038/bcj.2016.95

**Published:** 2016-11-04

**Authors:** V S Tompkins, R Sompallae, T R Rosean, S Walsh, M Acevedo, A L Kovalchuk, S-S Han, X Jing, C Holman, J E Rehg, S Herms, J S Sunderland, H C Morse, S Janz

**Affiliations:** 1 Department of Pathology, Iowa Institute of Human Genetics, University of Iowa Roy J. and Lucille A. Carver College of Medicine, Iowa City, IA, USA; 2 Bioinformatics Division, Iowa Institute of Human Genetics, University of Iowa Roy J. and Lucille A. Carver College of Medicine, Iowa City, IA, USA; 3 Department of Radiology, University of Iowa Roy J. and Lucille A. Carver College of Medicine, Iowa City, IA, USA; 4 Virology and Cellular Immunology Section, Laboratory of Immunogenetics, National Institute of Allergy and Infectious Diseases, National Institutes of Health, Rockville, MD, USA; 5 Department of Pathology, St Jude Children's Research Hospital, Memphis, TN, USA; 6 International Waldenstrom's Macroglobulinemia Foundation, Sarasota, FL, USA

## Abstract

Waldenström macroglobulinemia (WM) is a low-grade incurable immunoglobulin M^+^ (IgM^+^) lymphoplasmacytic lymphoma for which a genetically engineered mouse model of *de novo* tumor development is lacking. On the basis of evidence that the pro-inflammatory cytokine, interleukin 6 (IL6), and the survival-enhancing oncoprotein, B cell leukemia 2 (BCL2), have critical roles in the natural history of WM, we hypothesized that the enforced expression of IL6 and BCL2 in mice unable to perform immunoglobulin class switch recombination may result in a lymphoproliferative disease that mimics WM. To evaluate this possibility, we generated compound transgenic BALB/c mice that harbored the human *BCL2* and *IL6* transgenes, EμSV-BCL2-22 and H2-L^d^-hIL6, on the genetic background of activation-induced cytidine deaminase (AID) deficiency. We designated these mice BCL2^+^IL6^+^AID^−^ and found that they developed—with full genetic penetrance (100% incidence) and suitably short latency (93 days median survival)—a severe IgM^+^ lymphoproliferative disorder that recapitulated important features of human WM. However, the BCL2^+^IL6^+^AID^−^ model also exhibited shortcomings, such as low serum IgM levels and histopathological changes not seen in patients with WM, collectively indicating that further refinements of the model are required to achieve better correlations with disease characteristics of WM.

## Introduction

Waldenström macroglobulinemia (WM) is a low-grade lymphoplasmacytic lymphoma (LPL) associated with a monoclonal immunoglobulin M (mIgM) in the serum. LPL is composed of a mixture of malignant B-cells whose differentiation status ranges from small B lymphocytes to mature plasma cells.^1^ Prominently included is a fraction of B cells with intermediate cytological features, designated lymphoplasmacytic cells.^2^ LPL does not always lead to WM because it produces, in ~5% of cases, either a mIg that is not of the M class (IgA>IgG) or no Ig at all (non-secretory variant). Conversely, a serum ‘IgM spike' is not always caused by LPL because other B-lineage tumors including marginal zone B-cell lymphoma^3^ and, in rare cases, IgM myeloma^4^ are also associated with the laboratory finding. In summary, even though LPL does not always lead to WM and the detection of a serum IgM paraprotein is not pathognomonic for the disease, WM is always caused by IgM^+^ LPL.

Despite unprecedented progress in elucidating the natural history of WM,^5^ our understanding of the disease remains superficial—particularly with regard to etiology and genetic predisposition,^6^ the precise nature of the precursor cell^7^ and the molecular pathway of its malignant transformation.^8^ Likewise, despite significant recent improvements in treatment options for patients with WM,^9^ a complete remission is rarely achieved and the neoplasm remains incurable in the great majority of cases.^10^ Further therapeutic advances and the closure of pathophysiological knowledge gaps may depend in no small measure on the development of an accurate, genetically engineered mouse model (GEMM) of human IgM^+^ LPL in which WM-like neoplasms develop predictably with short latency and high tumor incidence.^11^

With that goal in mind and with evidence in hand that the pro-inflammatory cytokine, interleukin 6 (IL6), and the survival-enhancing oncoprotein, B cell leukemia 2 (BCL2), have important roles in the biology and genetics of WM,^12, 13, 14, 15^ we hypothesized that the enforced expression of IL6 and BCL2 in mice unable to undergo Ig class switch recombination (CSR) might be a useful first step toward designing a GEMM of human IgM^+^ LPL. Thus, we generated compound transgenic mice that harbored the human *BCL2* transgene, EμSV-BCL2-22 ^16^ (henceforth called BCL2^+^), and the human *IL6* transgene, H2-L^d^-hIL6 ^17^ (IL6^+^), on the plasmacytoma susceptible background of BALB/c (C) ^18^—additionally rendered deficient in activation-induced cytidine deaminase (AID) due to homozygosity for a null allele of the AID-encoding gene, *Aicda* (AID^−^).^19^ Based on our previous experience with tumor induction studies in BCL2^+^,^20^ IL6^+^^21, 22^ and AID^−^^23^ mice, we postulated that the newly generated strain, henceforth called BCL2^+^IL6^+^AID^−^, may be prone to IgM^+^ lymphomas that recapitulate important features of human WM.

Here we show that this expectation was met in some but not all respects. For example, although IgM^+^ lymphoproliferation including LPL-like neoplasia was fully penetrant in BCL2^+^IL6^+^AID^−^ mice, serum IgM levels were low compared with patients with WM and serum IgM spikes were rarely seen. Overcoming these deficiencies may require introduction of the hallmark WM *MYD88*^L265P^ point mutation^24^ to the BCL2^+^IL6^+^AID^−^ model, which may be accomplished by crossing in a newly developed conditional transgene harboring this mutation.^25^ A complementary approach may be lentiviral gene transduction of BCL2^+^IL6^+^AID^−^ B-cells with *CXCR4*^WHIM^ mutant alleles^26, 27^ followed by adoptive transfer (AdT) of the modified B cells to a suitable host in which lymphoma formation takes place—as recently shown for AdT models of human myeloma.^28, 29, 30^

## Materials and methods

### Mice

Compound transgenic BCL2^+^IL6^+^AID^−^ mice carry two dominant oncogenes, EμSV-BCL2-22 (BCL2^+^)^16^ and H2-L^d^-IL6 (IL6^+^)^17^ on the genetic background of BALB/c (C). In addition, the mice are homozygous for a null allele of the AID-encoding gene, *Aicda* (AID^−^).^31^ BCL2^+^IL6^+^AID^−^ mice were bred according to the scheme in Supplementary Figure 1a. This involved several intermediate strains, including BCL2^+^AID^−^ and IL6^+^AID^−^, used as controls. Genotyping relied on PCR (Supplementary Figure 1b). Mice were housed in the University of Iowa (UI) Animal Resource Center. All procedures involving mice were approved under IACUC Protocol 0701007.

### Histopathology and immunohistochemistry

At necropsy, a standard panel of tissues, including lymphoid organs (lymph nodes and spleen) and parenchymatous organs (liver, kidney), was harvested, fixed in formalin and embedded in paraffin. Tissue sections (4 μm) were deparaffinized, rehydrated and stained with hematoxylin and eosin. For immunohistochemistry, specimens were labeled for 2 h in blocking buffer (2.5% BSA, 5% goat serum, and 0.3% Triton X-100 in PBS) using antibodies described in the Supplementary Material and Methods section. Immunoreactivity was visualized using the Vecta ABC kit and DAB reagents (Vector Laboratories).

### Molecular, cytogenetic and flow cytometric tumor studies

Global gene expression profiling (GEP) relied on Illumina Mouse WG-6 v2.0 (San Diego, CA, USA) Bead Chips and data analytical approaches described elsewhere.^32^ NFκB DNA-binding activity was determined with the help of EMSA.^33^ Western blotting followed a recently published protocol^34^ using antibodies listed in Supplementary Material and Methods. Capillary-based Sanger sequencing of *Myd88*^L252^ and flanking regions relied on an ABI 3730xl (Thermo Fisher Scientific, Foster City, CA, USA) instrument provided by the HCCC Genomics Core. FISH was performed on metaphase chromosomes using gene-specific probes for *Igh* and *Myc*.^23^ Images were acquired using a DMRXA epifluorescence microscope equipped with a Sensys charge-coupled device camera (Roper Scientific, Trenton, NJ, USA). Surface expression of B cell and plasma cell markers was analyzed with the help of a FACSCanto II flow cytometer (Becton Dickinson (BD), San Jose, CA, USA)^35^ and antibodies in Supplementary Material and Methods. Non-specific Ab binding was blocked using rat serum (Jackson Immunoresearch, West Grove, PA, USA) and 10 μg 2.4G2 (BioXCell, West Lebanon, NH, USA). Data were analyzed using FlowJo (Tree Star, Ashland, OR, USA).

### Serum immunoglobulins, viscosity, cytokines and chemokines

Serum immunoglobulin levels were analyzed by ELISA.^36^ Whole blood was collected from mice at necropsy, using heart puncture. Blood samples were centrifuged at 14 000 r.p.m. for 5 min. After centrifugation, serum was removed and frozen until the time of analysis. Searchlight Arrays (Aushon Biosystems, Billerica, MA, USA) were used to determine cytokine and chemokine levels. Serum viscosity measurements relied on a calibrated laboratory capillary viscometer (UI Diagnostic laboratories service (Iowa City, IA, USA).

### FDG-PET/CT analysis

Integrated ^18^F-fluorodeoxyglucose positron emission tomography (FDG-PET) and computed tomography (CT) scanning of mice relied on an Inveon small-animal PET/CT/SPECT imaging system (Preclinical Solutions, Siemens Healthcare Molecular Imaging, Knoxville, TN, USA), as previously described.^37^ Images were analyzed using PMOD v3.2 software (PMOD Technologies, Zurich, Switzerland). To better appreciate tumor contours in anatomical context, 3D reconstructions of PET and CT modalities were generated using independent software (Inveon Research Workplace, Siemens).

## Results

### AID deficiency on the genetic background of C accelerates a BCL2^+^IL6^+^-driven B-cell disorder

**Figure 1:**
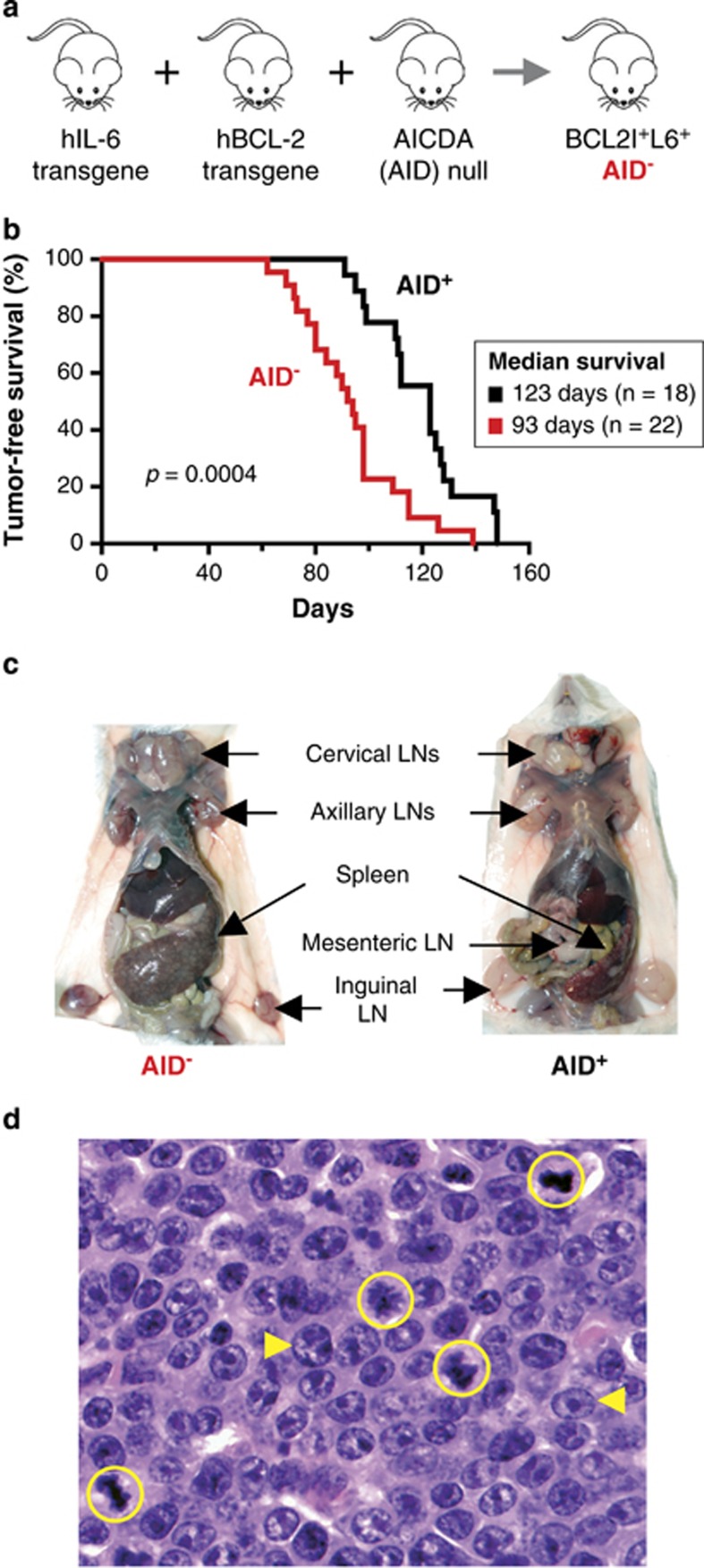
Manifestation and histopathological features of lymphoproliferative disease in BCL2^+^IL6^+^AID^−^ mice. (**a**) Generation of BCL2^+^IL6^+^AID^−^ mice. The human *BCL2* transgene EμSV-BCL2-22 (BCL2^+^) and human *IL6* transgene H2-L^d^-hIL6 (IL6^+^) were transferred to the *Aicda* null background of AID deficiency (AID^−^) using the breeding scheme depicted in Supplementary Figure 1. AID-proficient, double-transgenic BCL2^+^IL6^+^AID^+^ mice, generated by intercrossing strains BCL2^+^ and IL6^+^, were used as controls. Both transgenes and the *Aicda* null allele were on the genetic background of BALB/c (C), which is highly susceptible to malignant plasma cell transformation. (**b**) Kaplan–Meier curve showing tumor free survival of AID-deficient BCL2^+^IL6^+^AID^−^ mice (red curve) and AID-proficient BCL2^+^IL6^+^AID^+^ mice (black curve). (**c**) Necropsy photographs of a diseased BCL2^+^IL6^+^AID^−^ (left) and BCL2^+^IL6^+^AID^+^ (right) mouse. Some grossly enlarged lymph nodes (LNs) are labeled. (**d**) Photomicrograph of BCL2^+^IL6^+^AID^−^-dependent lymphoproliferation after staining with hematoxylin and eosin (H&E; original magnification 63x). Mitotic figures are circled yellow. Aberrant centroblast-like and immunoblast-like B cells are indicated by yellow arrowheads pointing right and left, respectively.

To determine whether enforced transgenic expression of BCL2 and IL6 acts synergistically with loss of activation-induced cytidine deaminase (AID) to promote the expansion and malignant transformation of IgM-producing B lymphocytes, AID-deficient BALB/c (C) BCL2^+^IL6^+^AID^−^ mice were monitored at weekly intervals for signs of tumor development, such as declining health status parameters and peripheral lymph node enlargements (Figure 1a). Twenty-two of 22 (100%) mice developed advanced disease requiring euthanasia for humane reasons at a median age of 93 days (mean 93.3±19.0 days; range 62–139 days; Figure 1b, red curve). Disease progression in an AID-proficient control group of BCL2^+^IL6^+^AID^+^ mice (*n=*18) was significantly slower (median survival 123 days; mean survival 120±17.6 days; range 91–148 days) based on Mantel-Cox log-rank analysis (*P*=0.0004; Figure 1b, black curve). Accelerated disease onset in the AID^−^ cohort was somewhat surprising given that, in agreement with the well-established mutagenic role of AID,^38^ loss of AID function has been demonstrated to inhibit malignant B-cell development in mice.^23, 39^ Supplementary Figure 2 shows that the AID^−^-dependent promotion of BCL2^+^IL6^+^-driven disease was attributable to the strong genetic collaboration of AID^−^ with enforced expression of BCL2 (*P*<10^−4^), overriding the opposing but relatively weak interaction of AID^−^ with transgenic expression of IL6 (*P*=0.0591). The result depicted in Figure 1b demonstrated the complete genetic penetrance of BCL2^+^IL6^+^-driven disease regardless of AID status and indicated that AID functions like a classic tumor suppressor in this specific genetic context.

### Most BCL2^+^IL6^+^AID^−^ mice succumb to lymphoproliferation, not frank lymphoma

Gross pathological assessment at necropsy revealed pronounced splenomegaly and lymphadenopathy in all mice included in Figure 1b (Figure 1c). Irrespective of AID status, the mean spleen weight was increased more than 10-fold: in AID-deficient mice (1.74±0.74 g; *n=*18) more severely (by ~50%) than in AID-proficient mice (1.15±0.74 g; *n=*18; *P*<0.05, Mann-Whitney *t* test). Histological analysis of hematoxylin and eosin-stained tissue sections of AID-deficient mice revealed a mixture of aberrant B lymphocytes, including large numbers of germinal center (GC)-like cells (centroblasts and immunoblasts), immature plasma cells (plasmablasts) and fully differentiated plasma cells. A representative high-power image of this cell population is shown in Figure 1d, using an abdominal lymph node as the example. Supplementary Figure 3a depicts low-power images of the same node, demonstrating that incipient lymphomas were surrounded by hypertrophic B-cell follicles identified by immunostaining for PAX5. A peripheral lymph node from a different case demonstrated another consistent feature of lymphoproliferation in AID-deficient mice: aggregates of aberrant CD138^+^ plasmablasts and plasma cells in the vicinity of enlarged follicles (Supplementary Figure 3b). The expanded population of these cells in the medullary areas of the lymph nodes and red pulp of the spleen (not shown) may represent extrafollicular accumulations that develop without the requirement for germinal center passage—as seen in tissues of autoimmune mice^40^ and likely promoted by IL6.^41^ On the basis of criteria described in the Bethesda classification of lymphoid neoplasms in laboratory mice,^42^ the histopathological changes seen in BCL2^+^IL6^+^AID^−^ mice were classified as a novel type of B-lymphoproliferative disorder that generates LPL-like lesions. However, the apparent lack of transplantability from these lesions together with PCR-based evidence that B-cell expansion was polyclonal in the majority of cases (results not shown) indicated that transformation to fully malignant B cells was incomplete. Thus, most BCL2^+^IL6^+^AID^−^ mice expire due to lymphoproliferative disease before frank lymphoma is manifested.

### BCL2^+^IL6^+^AID^−^ mice harbor elevated serum IgM levels but lack IgG and IgA

**Figure 2:**
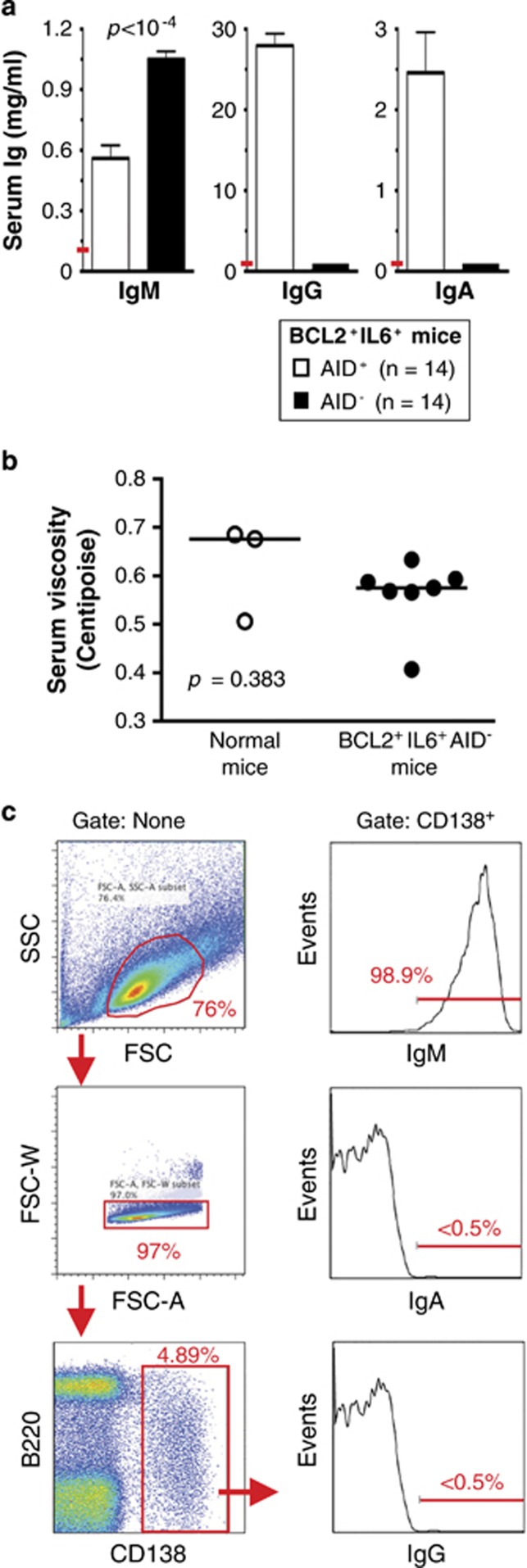
BCL2^+^IL6^+^AID^−^ mice bearing enlarged lymphoid tissues contain increased amounts of serum IgM and express IgM on the surface of B cells. (**a**) Elevated IgM (1050±39 μg/ml) but not IgG and IgA in the blood serum of BCL2^+^IL6^+^AID^−^ mice (black bars). Tumor-bearing BCL2^+^IL6^+^AID^+^ mice (white bars) exhibited elevations of all 3 isotypes; that is, mean IgM (560±65 μg/ml), IgG (27.9±1.49 mg/ml) and IgA (2.46±0.502 mg/ml) were 5.6, 140 and 276 times higher than the corresponding values in normal age-matched BALB/cByJ mice. The normal reference values, taken from The Jackson Laboratory's *Mouse Phenome Database* (Bar Harbor, ME, USA), are indicated by short red lines in the graphs' y-axes: IgM, ~100 μg/ml; IgG, ~200 μg/ml; IgA, ~8.9 μg/ml. (**b**) Unchanged serum viscosity in diseased BCL2^+^IL6^+^AID^−^ mice (0.561±0.0717 centipoise, *n=*7, closed circles) compared with age-matched normal C mice (0.622±0.101 centipoise, *n=*3, open circles). Measurements relied on a standard laboratory viscometer. (**c**) Flow cytometric analysis of surface IgM expression on B cells. Forward scatter (FSC) and side scatter (SSC) gated splenocytes were labeled with antibodies to the B-cell marker B220 and the plasma cell marker CD138 in order to identify B220^+^CD138^+^ plasmablasts and B220^−^CD138^+^ plasma cells (left panels). Staining of CD138^+^ cells with antibodies to Ig heavy chains revealed immunoreactivity to IgM, but not to IgG or IgA (right panel).

ELISA was used to determine whether BCL2^+^IL6^+^AID^−^ mice exhibited changes in serum Ig patterns that mimic the hyper-IgM syndrome of patients with WM. Serum samples (*n=*14) contained significant amounts of IgM (1.05 g/l on average) and, as expected, essentially no IgG and IgA (Figure 2a). In contrast, IgG and IgA were markedly elevated in BCL2^+^IL6^+^AID^+^ mice (*n=*14). Although mean IgM in BCL2^+^IL6^+^AID^−^ mice was ~10-fold higher than in normal age-matched controls (~0.1 g/l), the absolute levels of IgM were modest compared with patients with WM.^26^ This was consistent with both the absence of serum M-spikes in the great majority of mice (Supplementary Figure 4) and normal serum viscosity (0.561 centipoise on average) relative to the age-matched normal mice used as controls (0.622 centipoise; Figure 2b). In agreement with the ELISA results, flow cytometric evaluation of CD138^+^ plasmablasts and plasma cells from an enlarged lymph node of a BCL2^+^IL6^+^AID^−^ mouse demonstrated surface expression of pre-switch μ heavy chain but lack of post-switch α or γ chains (Figure 2c). The analysis of four lymphoid tissues (spleen and cervical, axillary and inguinal lymph nodes) from two additional mice demonstrated the remarkable consistency of IgM expression (Supplementary Figure 5); that is, even on the background of massive BCL2^+^IL6^+^-driven B-cell expansion, AID^−^ was not ‘leaky' in its ability to abrogate Ig class switch recombination.

### GEP of IgM^+^ lymphoproliferative disease points to MYD88/NFκB activation

**Figure 3:**
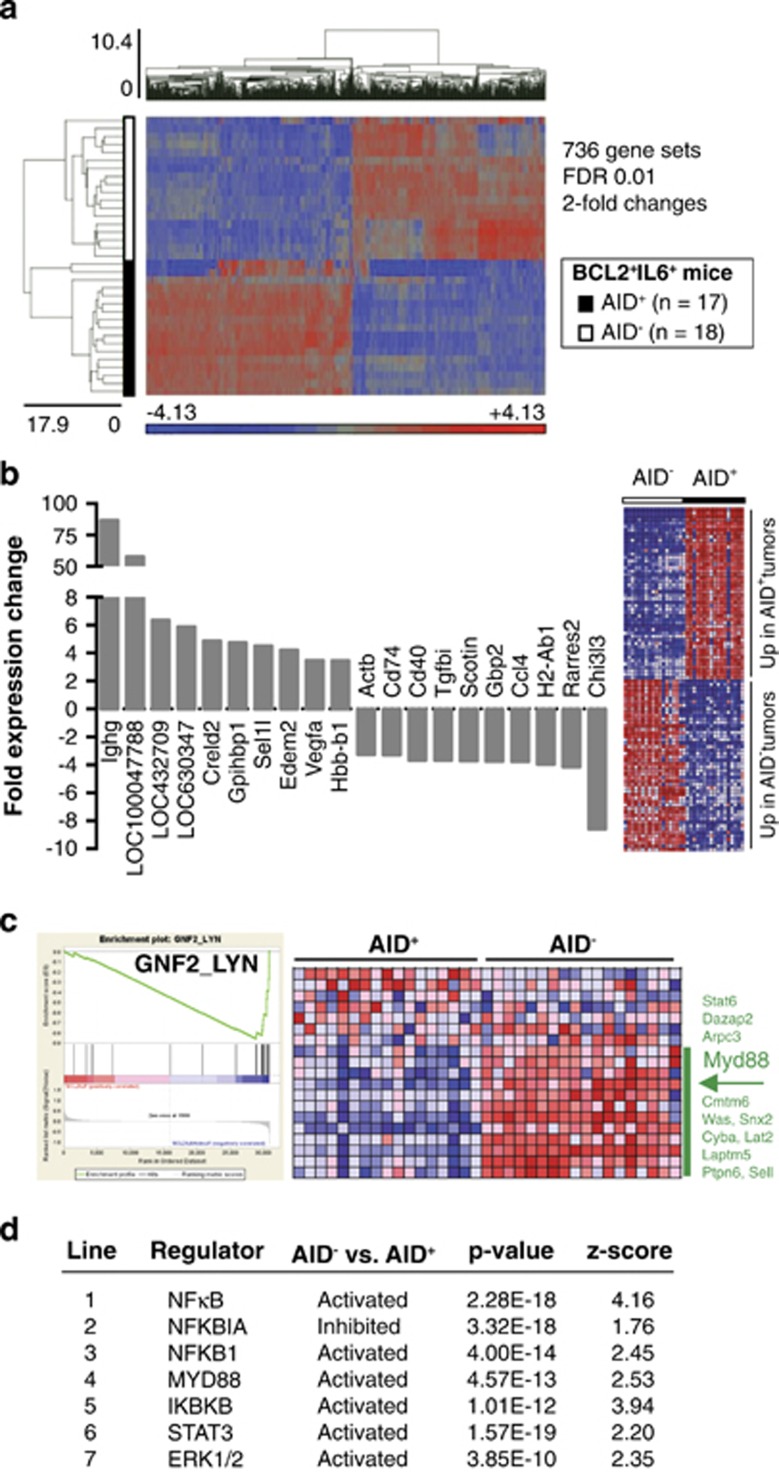
Gene expression profile of lymphoproliferative disease of BCL2^+^IL6^+^AID^−^ mice. (**a**) Heat map of differentially expressed genes in AID-deficient tissues (open square) compared with AID-proficient tissues (closed square). Up and downregulated genes are indicated in red and blue, respectively. Illumina microarray data were processed using GenomeStudio v1.9.0 (Illumina). Raw expression values were normalized using the quantile method and assessed for genes with differential expression based on analysis of variance (ANOVA). Probe set values were normalized to a mean of zero (mean centered) and a standard deviation of one. The false discovery rate (FDR) was then applied to adjust *P-*values for Multiple test correction. Differentially expressed genes were selected with thresholds based on FDR-adjusted *P-*values smaller than 0.01 and fold changes larger than 2 or smaller than −2. (**b**) Shown on the right is a heat map of the top-50 ANOVA-selected genes found to be differentially expressed in the AID^+^ and AID^−^ samples included in panel a. Shown on the left is a bar graph of fold expression changes of the top 10 up or downregulated genes (labeled below and above the x-axis, respectively). (**c**) Presented to the left is a gene set enrichment analysis-generated enrichment plot of a genetic pathway, GNF2_LYN, that distinguished AID^+^ and AID^−^ samples and contained *Myd88* as a core enrichment gene up-regulated in the AID^−^ sample. Depicted to the right is a heat map of the expression of GNF2_LYN enrichment genes in AID^+^ and AID^−^ tissues. The enrichment core (12 genes) is indicated by a green vertical line that is labeled with gene symbols. The location of *Myd88* (4th gene from the top) is marked by a left-pointing black arrow. (**d**) Table of pathway regulators (column 1) identified as activated or inhibited in AID^−^ samples (column 2) using IPA's upstream analysis module (Ingenuity Systems, Redwood City, CA, USA). The WebGestalt gene set analysis tool (http://bioinfo.vanderbilt.edu/webgestalt) confirmed the activation of NFκB in AID^−^ vs AID^+^ tumors, as NFκB was the only pathway represented twice among the top 10 pathways (not shown). The enrichment ratios were 3.61 (*P*=7.3 × 10^−5^) and 3.55 (*P*=2 × 10^−3^), respectively.

Microarray-based GEPs of lymphoid tissues from AID-deficient BCL2^+^IL6^+^ AID^−^ mice (*n=*18) and AID-proficient BCL2^+^IL6^+^AID^+^ mice (*n=*17) were used to glean insights into the genetic network of IgM^+^ lymphoproliferation. Analysis of variance and multi-test correction revealed 736 differentially expressed gene sets, using a cutoff of ⩾2-fold expression change and a false discovery rate (FDR) of ⩽1% (Figure 3a). The top 50 genes differentially expressed in AID^+^ vs AID^−^ samples are presented as a heat map map in Figure 3b, right. Unsurprisingly, genes over-expressed in AID^+^ included post-switch Ig heavy chain genes, such as *Ighg*, which was 135-fold elevated compared with AID^−^, and LOC100047788 (secreted γ2a heavy chain), which was 80-fold elevated. Prominently upregulated in AID^−^ samples were immune genes, including the B-lymphocyte differentiation marker *Cd40*, the MHC class II invariant chain CD74, and the chemokine *Ccl4* (Figure 3b, left). Gene set enrichment analysis and Ingenuity pathway analysis (IPA) were employed to interrogate the genetic network of IgM^+^ lymphoproliferation in greater depth. Gene set enrichment analysis implicated GNF2_LYN (enrichment plot shown in Figure 3c, left), a pathway that included *Myd88* among core genes upregulated in AID^−^ (indicated by a labeled horizontal arrow in Figure 3c, right). IPA's upstream analysis module confirmed the involvement of MYD88 as a proximal activator in AID^−^ (Figure 3d, line 4) and additionally demonstrated activation of STAT3 (downstream of IL6) and ERK signaling (lines 6-7), activation of the positive NFκB regulators, NFκB complex (line 1), NFKB1 (line 3) and IKBKB (line 5) and inhibition of the negative NFκB regulator, NFKBIA (line 2). This result provided genetic evidence for the involvement of MYD88/NFκB signaling in the mechanism by which BCL2^+^IL6^+^ promotes lymphoproliferation and lymphoma in AID^−^ mice.

### NFκB activation is not caused by WM-typical *Myd88*^L252^ mutation

**Figure 4:**
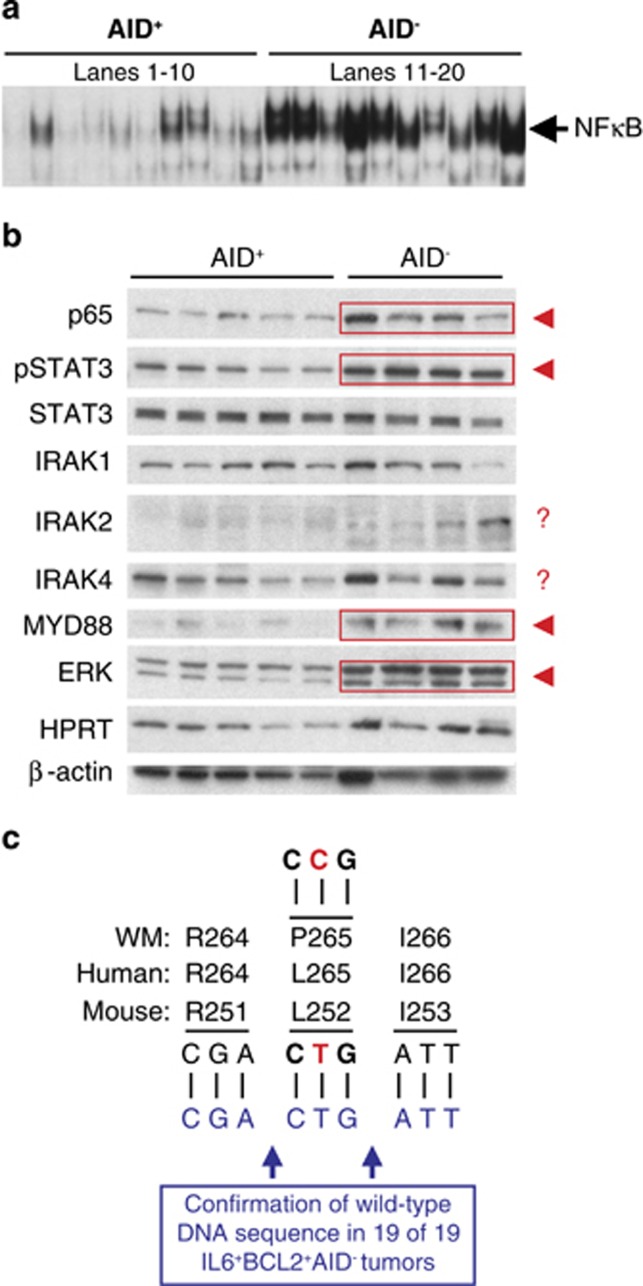
Activation of NFκB/STAT3 signaling in hyper- and/or neoplastic lymphoid tissues of BCL2^+^IL6^+^AID^−^ mice. (**a**) EMSA demonstrating heightened NFκB DNA-binding activity in AID^−^ tissues (*n=*10, lanes 11–20) compared with AID^+^ tissues (*n=*10, lanes 1-10). Competition studies ascertained the result, as incubation of nuclear extracts with 30-fold excess unlabeled competitor probe abolished the constitutive NFκB activity, whereas incubation with unlabeled probe containing a mutation that disabled NFκB binding did not (results not shown). (**b**) Western blotting of Myd88/NFκB/Stat3 pathway proteins in 5 AID^+^ (lanes 1–5) and 4 AID^−^ (lanes 6-9) tissues. Increased protein levels in AID^−^ samples (indicated by red arrowheads) were seen in case of the NFκB transcription factor p65, Erk, pStat3 and Myd88. Indicated by red question marks are putative increases in AID^−^ specimens that exhibited significant sample-to-sample variability. Increased expression of Myd88/p65, in conjunction with Stat3 activation, may reflect the signaling crosstalk that we previously observed in malignant, Myc-transgenic B cells.^33^ (**c**) Sanger sequencing of mouse *Myd88*^L252^ and flanking regions. The leucine-encoding mouse CTG codon 252 corresponds to the human 265 codon, *MYD88*^L265^. The wild-type status of *Myd88*^L252^ was confirmed in 19 of 19 tumor specimens from BCL2^+^IL6^+^AID^−^ mice (represented in blue at the bottom). Thus, there was no evidence for the leucine-to-proline substitution, L265P, which is caused by a highly recurrent T-to-C mutation in human WM.

EMSA was used to determine the IκB kinase (IKK)-dependent NFκB DNA-binding activity in 10 enlarged lymphoid tissues each of AID^+^ and AID^−^ mice. Despite considerable sample-to-sample variability in both groups, the average NFκB DNA-binding activity was significantly elevated in the AID^−^ samples (Figure 4a). In accordance with that, immunoblotting of five AID^+^ and four AID^−^ tissues revealed increased amounts of the NFκB transcription factor, p65, in the former (Figure 4b, top panel, indicated by red rectangle and arrowhead). In addition, AID^−^ samples contained increased levels of pSTAT3, ERK and MYD88 by comparison with their AID^+^ counterparts. AID-dependent differences in interleukin-1 receptor associated kinases (IRAK1, 2 and 4) and TAK1 (TGF-β activated kinase 1 a.k.a. NR2C2, result not shown)—critical pathway regulators downstream of MYD88 and upstream of IKK—were not observed (Figure 4b). These findings suggested that the NFκB/STAT3 axis is activated in BCL2^+^IL6^+^AID^−^ B cells, as previously seen in MYC-driven B-lymphomas.^33^ Considering that NFκB signaling is constitutively activated in human WM by virtue of the *MYD88*^L265P^ mutation, the findings also raised the question as to whether the same mutation might occur in the mouse model. Sanger sequencing of the corresponding mouse codon, *Myd88*^L252^, in 19 independent tissue specimens demonstrated that this was not the case (Figure 4c). Hence, elevated NFκB activity in the BCL2^+^IL6^+^AID^−^ model is not achieved by a WM-typical *Myd88* mutation.

### IgM^+^ lymphoproliferation undergoes major expansion at extra-medullary tissue sites

**Figure 5:**
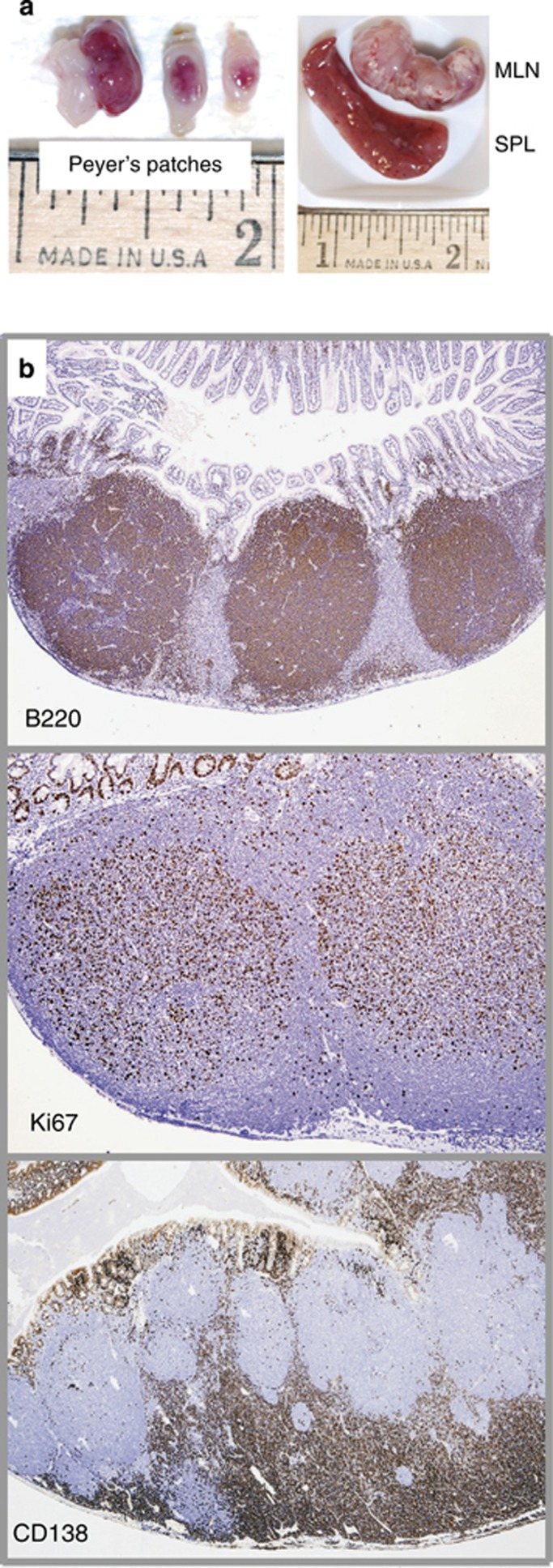
Gross and histopathology of lymphoid tissues of strain BCL2^+^IL6^+^AID^−^ mice. (**a**) Necropsy photographs of a representative disease-bearing mouse that contained multiple Peyer's patches at different stages of malignant development (left). Spleen (SPL) and mesenteric lymph node (MLN) of the same mouse were grossly enlarged (right). In some cases, MLNs reached weights of 3 grams. (**b**)**** Low-power photomicrographs of immunolabeled FFPE tissue sections of Peyer's patches at an early (top and center panels) or more advanced (bottom panel) stage of disease progression. Increased B220^+^ follicles (top) contain highly proliferative Ki67^+^ B cells (center), whereas interfollicular areas harbor large numbers of CD138^+^ lymphoplasmacytic cells (bottom). In some moribund mice, enlarged Peyer's patches seemed to be the underlying reason for intestinal obstructions and intussusceptions.

**Figure 6:**
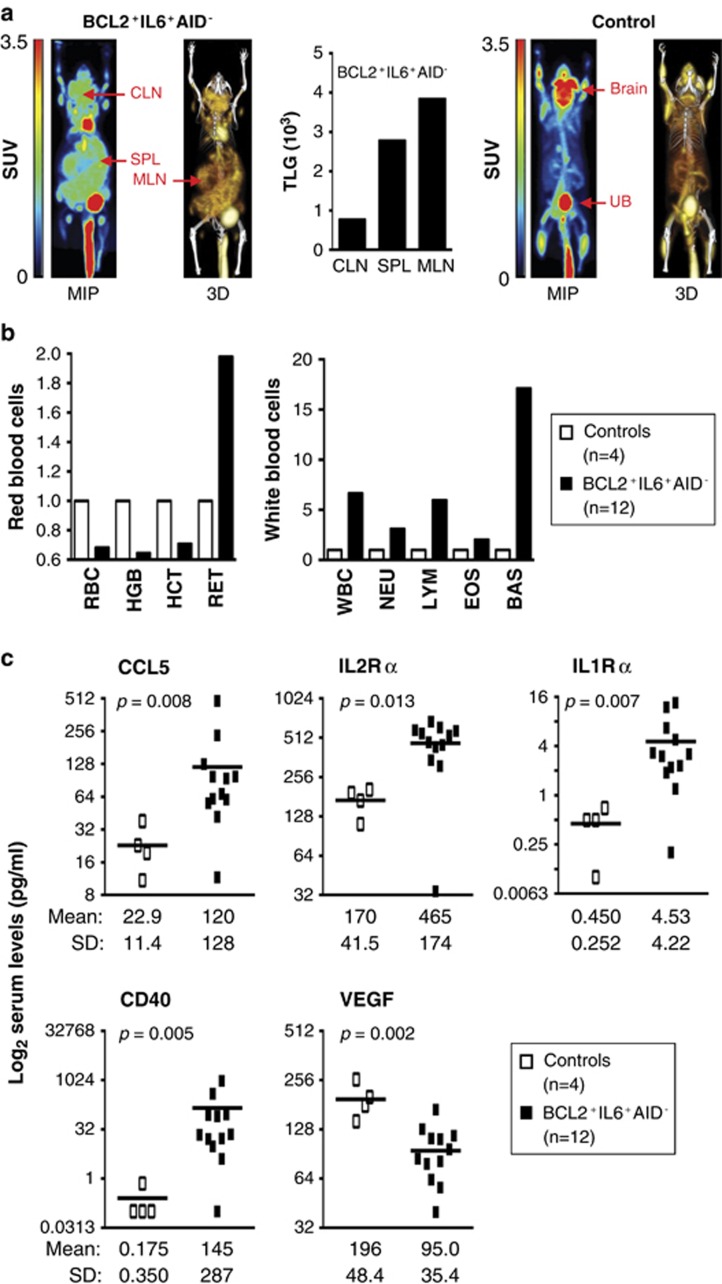
FDG-PET imaging and evaluation of changes in blood cells and serum cyto- and chemokines in disease-bearing BCL2^+^IL6^+^AID^−^ mice. (**a**) Comparison of maximal projection and 3D FDG-PET/CT images of a disease-bearing mouse (left) and normal mouse used as control (right). The whole-body disease burden was estimated at 35% in this case. Shown in the center is a bar graph of total lesion glycolysis (TLG) in cervical lymph node (CLN), spleen (SPL) and mesenteric lymph node (MLN). TLG, the product of maximal standardized FDG uptake and metabolic tumor volume, indicates the tumor's metabolic activity (glucose metabolism). (**b**) Changes in red and white blood cell parameters in disease-bearing mice (*n=*12, filled squares) and controls (*n=*4, open squares) detected with the help of a Sysmex XT veterinary hematology analyzer (Sysmex, Kobe, Japan). See Supplementary Table 1 for additional details. (**c**) Serum cyto- and chemokine levels significantly different in disease-harboring (*n=*12) and normal mice (*n=*4). Individual and mean values are represented by open/filled rectangles and horizontal bars, respectively. Mean values and standard deviations of the mean (SD) are indicated at bottom. Results of two-tailed *t*-tests (Mann–Whitney) are indicated. Gene ontology analysis of BCL2^+^IL6^+^AID^−^ tumors supported the findings shown here by virtue of indicating heightened activity of a cytokine and chemokine pathway (ID 0005125; 5.73 enrichment ratio; adjusted *P*=2.18 × 10^−7^) that included CCL5, other chemokines (CCL4, 7, 9 and 24; CXCL9, 10 and 12), interleukins (IL4 and 33), cytokines (CSF1 and MIF) and a transcription factor, SPP1, recently implicated in peritoneal plasmacytomagenesis in C mice.^32^

Histological examination of disease-bearing BCL2^+^IL6^+^AID^−^ mice (Figure 5a) invariably demonstrated enlarged lymphoid tissues containing hyperactive B220^+^ follicles and extensive extrafollicular accumulations of CD138^+^ lymphoplasmacytic cells. The changes are illustrated by Peyer's patches (Figure 5b) that harbored large B220^+^ follicles (top panel), with the majority of cells undergoing proliferation (based on immunoreactivity to Ki67; center) and CD138^+^ cells piling up in interfollicular areas (bottom). At time of sacrifice, all mice had progressed to a systemic dissemination pattern characterized by dense lymphoplasmacytic infiltrates in bone marrow, kidney, lung, liver (Supplementary Figure 6) and other organs. An unexpected but recurrent observation (five cases) was histiocytic sarcoma of the spleen (Supplementary Figure 7), a pathology not seen in patients with WM but reported for follicular lymphoma.^43^ Integrated FDG-PET and CT imaging^37^ was employed in three mice with advanced disease to measure the extent of lymphoproliferation in an objective and lesion-specific manner. Figure 6a, left, shows a maximum projection coronal PET image (MIP) and three-dimensional rendering of fused PET/CT images (3D) of one diseased mouse next to a control (right). The bar diagram in the center demonstrates that peak metabolic activity, determined as total lesion glycolysis (TLG), occurred in the mesenteric lymph node (Figure 6a, right; it occurred in the spleen in the two other cases as shown in Supplementary Figure 8). These results indicated that the IgM^+^ lymphoproliferative disease in BCL2^+^IL6^+^AID^−^ mice is a metabolically active process that results in massive lymphoid tissue expansion at extra-medullary sites.

### Disease-bearing BCL2^+^IL6^+^AID^−^ mice are anemic and exhibit serum cytokine changes seen in patients with WM

Clinical features of WM include changes of peripheral blood, such as anemia, leukocytosis and increased levels of cytokines and chemokines.^11^ Peripheral blood samples from BCL2^+^IL6^+^AID^−^ mice (*n=*12) demonstrated similar alterations (Supplementary Table 1), including anemia characterized by a decrease in red blood cells, hemoglobin and hematocrit and a compensatory increase in reticulocytes (Figure 6b, left). White blood cell counts revealed moderate increases in lymphocytes, neutrophils and eosinophils and a pronounced elevation of basophils—all consistent with features of leukocytosis seen in patients with WM (Figure 6, right). Following up on a landmark report on the cytokine and chemokine milieu in human WM,^15^ serum levels of 20 cyto- and chemokines were measured in BCL2^+^IL6^+^AID^−^ mice (*n=*12; Supplementary Table 2). Chemokine (C-C motif) ligand 5 (CCL5)—an important activator of the IL6/IgM axis in WM—was as highly upregulated in mice (5.24-fold) as in WM (Figure 6c). Similarly, just like in WM, in which soluble IL-2 receptor (sIL-2R) was among the five most highly elevated cytokines, the mice exhibited a nearly three-fold increase in IL-2Rα. Other cytokines exhibited the same trend in humans and mice, but the extent was different; for example, mice demonstrated a highly significant, ~10-fold increase in soluble IL-1 receptor alpha, whereas WM patients showed only a small increase (not significant). These results established important parallels between the lymphoid tissue microenvironment of the BCL2^+^IL6^+^AID^−^ model and the tumor microenvironment (TME) in human WM.

### Genetic pathways operating in aberrant BCL2^+^IL6^+^AID^−^ B-cells

**Figure 7:**
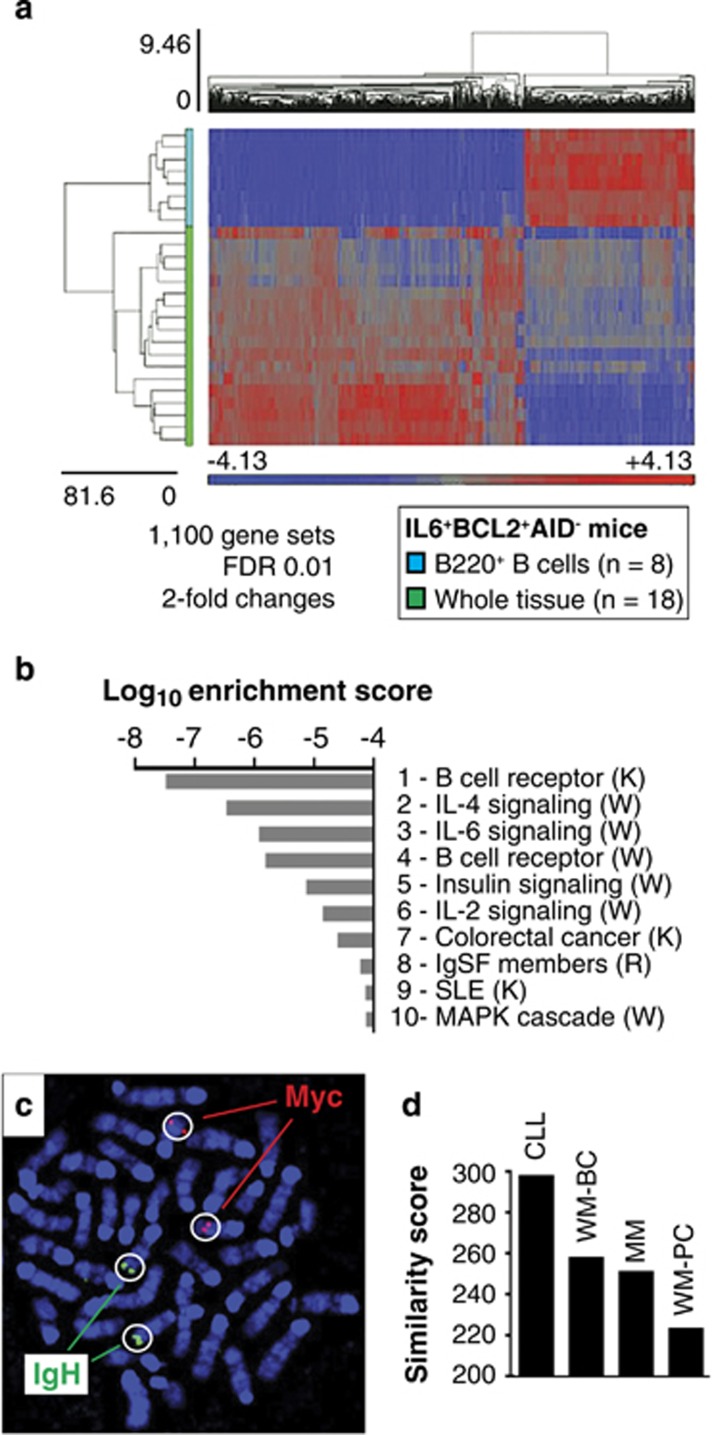
Genetic makeup of aberrant BCL2^+^IL6^+^AID^−^ B-cells. (**a**) Heat map of differentially expressed genes in fractionated B220^+^ B-cell (blue, *n=*8) vs intact WT (whole tissue) samples (green, *n=*18) obtained from disease-bearing BCL2^+^IL6^+^AID^−^ mice. Significantly up- and downregulated genes (red and blue, respectively) were determined as described in the legend of Figure 3a, using FDR 0.01, and fold change >2 or <−2. (**b**) Genetic pathway enrichment in B220^+^ cells identified by IPA. Online pathway repositories employed were as follow: K, KEGG W, Wikipathways; R, Reactome. Full designations of pathways 8 and 9 are IgSF family member proteins and systemic lupus erythematosus, respectively. (**c**) Representative metaphase spread of a tumor cell labeled with FISH probes annealing to the cellular oncogene, *Myc*, which resides on Chr 15 (red), or the Ig heavy-chain locus, *Igh*, on Chr 12 (green). Genetic recombination of *Myc* and *Igh*, which takes the form of a reciprocal chromosomal T(12;15) translocation, is a common feature of plasma cell tumors in C mice, providing the rationale for this analysis. (**d**) Bar graph diagram depicting genetic pathway similarity scores of mouse B-cells to human multiple myeloma (MM), chronic lymphocytic leukemia (CLL) and WM. The latter is represented by two different fractions: B cells (BC) and plasma cells (PC).

B-cell fractionation was combined with GEP to assess genetic pathways that specifically operate in BCL2^+^IL6^+^AID^−^ B cells or their microenvironment. Specimens of enlarged lymphoid tissues were halved, with one half being used for magnetic bead isolation of B220^+^ B-cells and determination of the ‘B-cell' GEP. The other half was left intact and used to determine the ‘whole-tissue' (WT) GEP. Comparison of 8 B-cell and 18 WT profiles uncovered 1110 differentially expressed gene probes with fold change ⩾2 at FDR 0.01 (Figure 7a). The top 10 genes up-regulated in B220^+^ cells included the important B-cell genes *Blk*, *Cd19* and *Cd22*, whereas the top 10 genes downregulated in this sample (up in stroma) encoded proteins that one might expect to function mainly in the microenvironment: LYZ (lysozyme), TIMP1 (metalloproteinase inhibitor), SERPING1 (extracellular matrix protein), ARG1 (arginase, an important immune modulator in macrophages), CHIA (chitinase) and RARRES2 (retinoic acid pathway; results not shown). Next, the rank-ordered GEP of the B220^+^ B-cell sample was subjected to gene set enrichment analysis using the KEGG, Wikipathways, Netpath and Reactome online repositories. This revealed B-cell receptor signaling (implicated twice) and IL4 and IL6 signaling as the top four activated pathways (Figure 7b). MYC (c-myc)-related pathways were conspicuously absent, but this was consistent with the lack of AID-dependent cytogenetic *Myc-Igh* rearrangement (chromosomal 12;15 translocation)^44, 45^ seen in three of three tumors (Figure 7c). Finally, cross-species genetic pathway likelihood scores^29^ were determined to quantify the relatedness of the BCL2^+^IL6^+^AID^−^ B cell to malignant B cells in humans. Figure 7d shows, firstly, that the mouse B-cells resembled the WM-derived B cells (WM-BC) more closely than the WM-derived plasma cells (WM-PC). Secondly, the similarity to CLL was greater than to myeloma—a reminder of the significant overlap of gene expression patterns of WM and CLL, discovered in the early days of WM genomics.^12^

### Genetic pathways governing the tissue microenvironment

**Figure 8:**
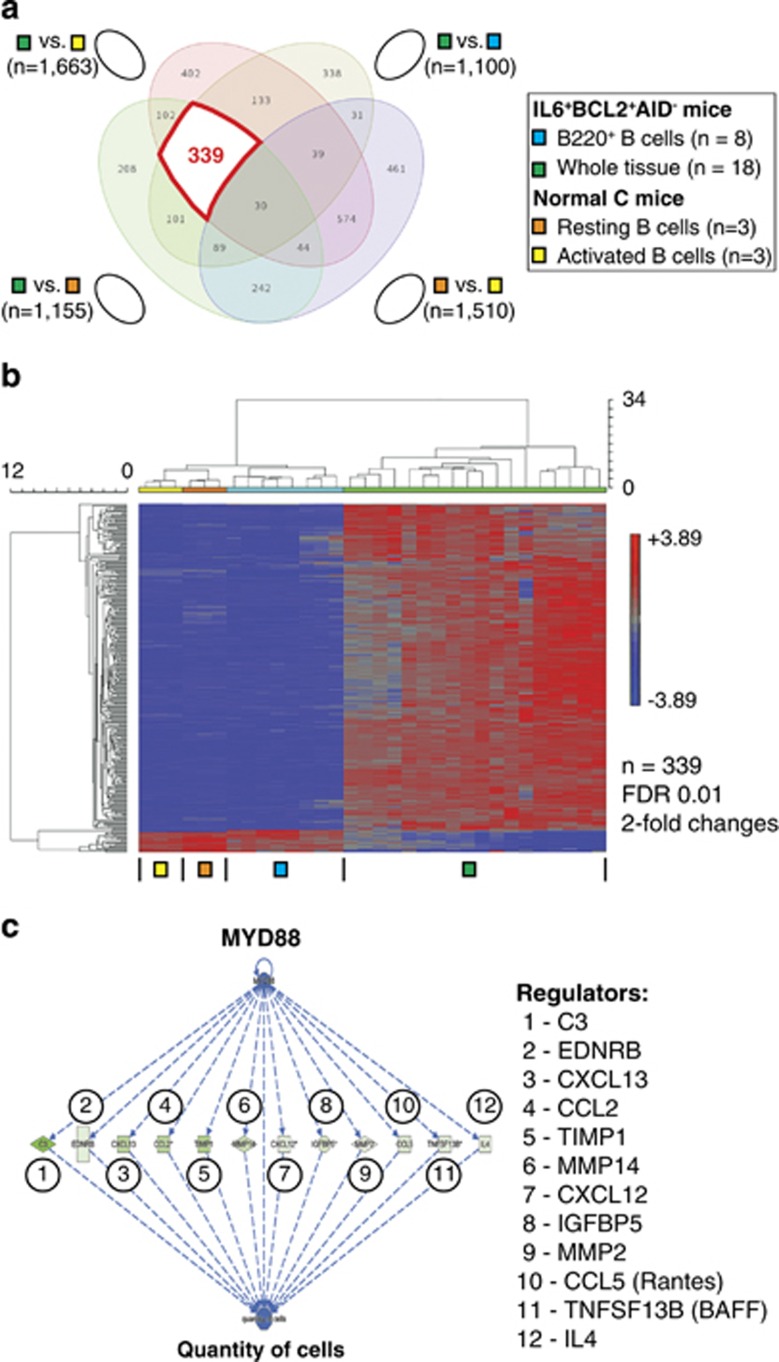
Genetic pathways in the BCL2^+^IL6^+^AID^−^ tissue microenvironment. (**a**) Venn diagram of partially overlapping, differentially expressed gene sets in normal and malignant B cells. The subset of annotated genes that is represented by 339 gene probes indicated by the thick red line constitutes the signature of the lymphoid tissue microenvironment in disease-bearing BCL2^+^IL6^+^AID^−^ mice. (**b**) Heat map of subset from panel a described above. (**c**) IPA upstream regulator analysis indicating activation of a MYD88-dependent network that targets 12 network members to control the ‘quantity of cells' in the tissue microenvironment. Genes upregulated are in green. Intensity of color is descriptive of the level of upregulation.

An indirect, subtractive approach was employed to evaluate the gene expression signature of the tissue microenvironment of BCL2^+^IL6^+^AID^−^ mice. The 1100 genes differentially expressed in WT and B-cell samples (Figure 7a) were compared with genes differentially regulated in WT vs normal resting (*n=*1155) and proliferating B cells (*n=*1663). A comparison of resting and activated B cells (1510 differentially expressed genes) was also included to facilitate the distinction of normal B-cell proliferation genes and genes operating in the microenvironment. The Venn diagram in Figure 8a, which depicts the overlap of the four pairwise comparisons described above, highlights a subset of 339 gene probes (indicated in red) that were common to all WT vs B-cell comparisons but absent in the normal proliferation signature. This subset is postulated to constitute the core signature of the tissue microenvironment of BCL2^+^IL6^+^AID^−^ mice. The heat map in Figure 8b demonstrates that the great majority of signature genes are up-regulated in WT samples but downregulated in B-cell samples. IPA placed many genes in canonical pathways tightly associated with stromal cell function: fibrosis, myeloid cell trafficking, inhibition of matrix metalloproteases, complement and immune and acute phase responses (Supplementary Figure 9a). T-cell pathways were also overrepresented in the IPA output (10 of 26 (40%) top pathways), suggesting that T-lymphocytes have an important role in shaping the BCL2^+^IL6^+^AID^−^ microenvironment. In addition, IPA's network analysis tool revealed the NFκB complex as a crucial network hub (Supplementary Figure 9b), whereas IPA's upstream regulator analysis tool identified MYD88 as the top proximal regulator in the TME (Figure 8c). These findings provided additional support for the contention that the tissue microenvironment of the BCL2^+^IL6^+^AID^−^ model shares important features with the TME of human WM.

## Discussion

The main finding of this study is the development of a mouse model of an IgM^+^ lymphoproliferative disorder that recapitulates important features of human WM. IgM^+^ lymphoproliferation and, in at least two cases with serum IgM M-spikes, incipient LPL-like neoplasms arose in BCL2^+^IL6^+^AID^−^ mice with full genetic penetrance (100% incidence) and relatively short latency (~3-month survival), progressed with a predominantly extra-medullar growth pattern (splenomegaly and lymphadenopathy) and exhibited molecular genetic traits (MYD88/NFκB activation) that overlapped with human WM. Although IgM^+^ lymphoproliferations and B lymphomas demonstrating lymphoplasmacytic features have been observed in laboratory mice in the past—for example, in C mice deficient in Fas signaling,^46, 47^ NSF.V^+^ mice congenic for an ecotropic murine leukemia virus^48, 49^ and mice in which the *Trp53*-encoded tumor suppressor, p53, had been specifically inactivated in mature B-cells^50^—none of these models mimic human WM as consistently as the BCL2^+^IL6^+^AID^−^ model. As such, the new strain of mice defines an important milestone towards the development of a faithful preclinical model system of WM pathogenesis. Strain BCL2^+^IL6^+^AID^−^ mice, which will be freely distributed to qualified investigators upon request, have been cryopreserved at The Jackson Laboratory (Bar Harbor, ME, USA) on behalf of the International Waldenstrom's Macroglobulinemia Foundation.

A surprising result of this investigation was the disease-promoting role of AID deficiency (AID^−^) in BCL2^+^IL6^+^ mice. Previous tumor induction studies using AID^−^ mice that carried a *Bcl2l1*^(ref. 23)^ or *BCL6*^(ref. 39)^ transgene demonstrated the opposite result; that is, AID deficiency slowed the development of B-cell cancers. An indication of decelerated oncogenesis was also observed in this study upon comparison of AID-deficient IL6^+^AID^−^ mice (303 days median survival) and AID-proficient IL6^+^AID^+^ mice (220 days, *P*=0.0591, Supplementary Figure 2a). However, this trend was overpowered by the striking AID^−^-dependent tumor acceleration seen upon comparison of BCL2^+^AID^−^ mice (216 days) and BCL2^+^AID^+^ mice (384 days, *P*<10^−4^). Thus, in compound BCL2^+^IL6^+^AID^−^ transgenics, AID^−^-dependent acceleration of BCL2^+^-driven lymphoproliferation appears to overcompensate for AID^−^-dependent deceleration of IL6^+^ driven lymphoproliferation. While it is possible that AID deficiency enhances the level of inflammation, possibly leading to a larger pool of replicating, long-lived, BCL2-transgenic lymphocytes, the underlying mechanism is not known. It may involve the special cytokine/chemokine milieu of BCL2^+^IL6^+^AID^−^ mice, other yet-to-be-elucidated features of the TME, or differences in precursor cells targeted for malignant transformation (for example, GC B-cells in case of AID^+^ vs pre-GC B cells in case of AID^−^). More work is needed to evaluate these possibilities.

The BCL2^+^IL6^+^AID^−^ model exhibits several shortcomings that should be acknowledged. First, among these is that it was designed as a phenocopy (not a genocopy) of human WM, with generation of IgM^+^ LPL-like tumors as the primary goal. Since this required abrogation of Ig isotype switching in tumor precursors, we took advantage of AID^−^ as a genetic engineering tool—fully recognizing that genetically-determined AID deficiency is not a feature of human WM. Another limitation of the mouse model concerns the low serum IgM level compared with WM. To put this into perspective, it may be useful to recall that, firstly, the mean serum IgM level in BCL2^+^IL6^+^AID^−^ mice (~1 g/l) was ~10 times higher than the reference level of age-matched controls (~0.1 g/l). Secondly, patients with WM exhibit the same 10-fold increase in serum IgM, except it occurs from a higher baseline: the upper limit of serum IgM in healthy individuals approaches 3 g/l.^51^ For example, a recent study reported that serum IgM levels in WM patients with untreated disease (*n=*102) or previously treated disease (*n=*62) averaged 26.7 g/l and 36.1 g/l, respectively.^26^ Thus, despite the same relative 10-fold increase in humans (from ~3 g/l to ~30 g/l) and mice (from ~0.1 g/l to ~1 g/l), mean serum IgM in the BCL2^+^IL6^+^AID^−^ model was ~30 times lower than in WM. Obviously, this renders the mouse model ill-suited for studies on serum hyperviscosity, tissue deposition of mIgM and other pathologies associated with hyper-IgM syndrome.

The above-described limitations notwithstanding, the newly developed BCL2^+^IL6^+^AID^−^ model defines a step forward in our ability to complement cell line-based preclinical studies on WM ^52, 53^ with studies on tumor development in laboratory mice. *In vivo* studies of this sort—under defined genetic, environmental and dietary conditions afforded by GEMMs of human cancer—may not only speed up the evaluation of new treatment options for patients with WM,^54, 55, 56, 57, 58^ but also permit us to close thorny knowledge gaps in our understanding of the natural history of the disease.^59^ New insights gleaned from integrated analyses of the WM genome^60^ will guide future approaches to improve on the BCL2^+^IL6^+^AID^−^ model.
