# Supplementary material for: Transgenic mouse model of IgM+ lymphoproliferative disease mimicking Waldenström macroglobulinemia
Source: Blood Cancer J. 2016 Nov 4;6(11):e488–. doi: 10.1038/bcj.2016.95 (PMC5148059; doi:10.1038/bcj.2016.95)
Supplement: Supplementary Information [file bcj201695x1.docx]

**Supplemental Material and Methods**

**Antibodies**

Antibodies used for immunohistochemistry (IHC) were the following: PAX5 (1H9, 649702), CD3 (145-2C11,100301), B220 (RA3 6B2,103201), Mac2 (M3/38,125401), CD138 (281-2,142501) from BioLegend and Ki67 (D3B5, 12202) from BD Biosciences. Western blotting relied on the following antibodies: p65 (C-20, sc-372), IRAK1, 2 and 4 (H-273, sc-7883; G-20,sc-23652 and T-16, sc-34470), HPRT (FL-218, sc-20975) and β-actin (C4,sc-47778) from Santa Cruz Biotechnology (Dallas, TX). pERK (197G2, #4377), ERK (#9102), pSTAT3 (#9131), STAT3 (#9132), MYD88 (D80F5, #4283), pTAK1 (90C7, #4508), TAK1 (D94D7, #5206) from Cell Signaling Technology (Danvers, MA). Antibodies for flow cytometry were as follows: B220-PE-Cy7 (RA3-6B2, 25-0452) and CD19-PE (1D3, 12-0193) from eBioscience (San Diego, CA); IgM-FITC (RMM-1, 406506), IgG-FITC (Poly4053, 405305) from Biolegend (San Diego, CA); IgA-FITC (C10-1, 556960) and CD138-APC (281-2, 558626) from BD.

**Supplemental Figure Legends**

**Supplemental Figure 1.** Overview of breeding scheme for generating BCL2^+^IL6^+^AID^-^ mice.

**(a)** Two strains of mice were newly developed at the beginning of this project: BCL2^+^AID^-^ and IL6^+^AID^-^. The mice were intercrossed to make compound BCL2^+^IL6^+^AID^-^ transgenics. Generation of IL6^+^AID^-^ mice involved two steps. The first relied on intercrossing heterozygous transgenic (TG) IL6 mice and homozygous TG AID^null^ mice, followed by the selection of IL6-TG offspring that carried one copy of an AID null allele (inherited from the AID-deficient AID^null^ parent) and one copy of an AID wild-type allele designated by “+” symbol (inherited from the AID-proficient IL6-TG parent). These offspring were observed at the expected Mendelian frequency of ~50%. The second step relied on the backcross onto AID^null^, followed by selection of IL6-TG pups homozygous for the AID null allele. Pups of this sort, designated IL6^+^AID^-^, occurred at a frequency of ~25%. The same 2-step strategy was used to make BCL2^+^AID^-^ mice. The intercross of IL6^+^AID^-^ and BCL2^+^AID^-^ at the bottom of the scheme resulted in 1 of 4 offspring (25%) with the desired genotype: BCL2^+^IL6^+^AID^-^. These mice were assembled in a study cohort and observed for tumor development as described in the main text.

**(b)** PCR-based genotyping of mice described in panel a took advantage of dedicated assays for the *BCL2* and *IL6* transgenes (top and center panel, respectively) and the AID-encoding *Aicda* wild-type and *Aicda* null allele (bottom panel). All 16 mice included here were homozygous for the *Aicda* null allele and, therefore, AID deficient (AID^-^). Mice 8 and 12 also carried the *BCL2* transgene: BCL2^+^AID^-^. Mice 5 and 10 also carried the *IL6* transgene: IL6^+^AID^-^. Mice 6, 9, 15 and 16 carried both transgenes: BCL2^+^IL6^+^AID^-^.

**Supplemental Figure 2.** Survival of AID-proficient (AID^+^) and AID-deficient (AID^-^) *BCL2* and *IL6* transgenic (TG) BALB/c mice (panel a) and gross pathological findings at necropsy in the AID^-^ mice (b and c).

**(a)** Tumor-free survival of *BCL2* TG mice (top) and *IL6* TG mice (bottom). AID status, number of mice and median survival of mice are indicated. Log rank analysis demonstrated a strikingly reduced survival of AID-deficient BCL2^+^ mice compared to AID-proficient counterparts (*p* < 0.0001). Mean survival including standard deviation and range were as follows: BCL2^+^AID^+^ (440 ± 102 d; range 327 – 623), BCL2^+^AID^-^ (205 ± 56.1 d; range 115 – 312), IL6^+^AID^+^ (240 ± 80 d; range 130 – 465), IL6^+^AID^-^ (299 ± 52.9 d; range 141 – 348).

**(b)** All genotypes included in panel a demonstrated at necropsy generalized splenolymphadenopathy. One representative image each from the *BCL2* and *IL6* cohort is shown. A photographic image of spleen and mesenteric lymph node is shown next to the mouse from which the tissues were obtained. Mean spleen weights in the BCL2^+^AID^-^ cohort (1.37 ± 0.89 g; range 0.15 – 2.9 g; n = 18) and IL6^+^AID^-^ cohort (1.13 ± 0.76 g; range 0.15 – 2.5 g; n = 14) were comparable.

**(c)** IL6^+^AID^-^ mice repeatedly presented with changes that were *not* seen in any other of the strains included in panel a. Changes included apparent circulatory and/or blood coagulation problems that resulted in dilated blood vessels in the sub-cutis (indicated by arrowheads in leftmost image), the mesentery (2^nd^ image) and many other tissue sites (not shown). Similar problems in the microcirculation may be the underlying reason for necrotic body tips including ears (3^rd^ image) and toes (not shown). Another peculiarity of strain IL6^+^AID^-^ was pronounced nodular lymphoid growth in the gut wall, leading to pearls on a chain-like structures as shown in the rightmost image.

**Supplemental Figure 3.** Histopathology of incipient lymphoma in BCL2^+^IL6^+^AID^-^ mice.

**(a)** Low-power photomicrograph (4X original magnification) of FFPE lymph-node sections immune labeled with antibody to Pax5 (top) or stained according to H&E (bottom). Enlarged, aberrant Pax5^+^ follicles surround an area of malignant B cells (circumscribed by red line) that, at higher power, is indistinguishable from the LPL-like tumor presented in Figure 1d of the main text.

**(b)** Shown in the upper panel is a low-power photomicrograph (4X original magnification) of a FFPE lymph-node section from another mouse, which has been immunolabeled with an antibody to the plasma-cell marker CD138 (syndecan 1). The lower panel shows at higher magnification (20X) that malignant plasma cells form sizeable aggregates in inter-follicular and medullary areas of the lymph node – in close proximity to enlarged B-cell follicles containing aberrant germinal centers.

**Supplemental Figure 4.**  Rare occurrence of serum IgM paraproteins in tumor-bearing BCL2^+^IL6^+^AID^-^ mice. Whole blood was collected from mice at necropsy, using heart puncture. Blood was transferred to EDTA-coated Microtainer tubes (Becton Dickinson) and spun for 5 min at 14,000 RPM to obtain serum. After centrifugation, serum was removed and frozen until the time of analysis. Serum protein electrophoresis was used to detect M-spikes. Serum proteins were fractionated on Hydragel Protein(e) K20 gels using a Sebia elecrophoresis chamber (90 V constant; 40 min migration time; 12±3 mA). Screening results of 12 mice are shown. Only one mouse (upper panel, lane 5) contained a prominent M-spike (indicated by red arrowhead), which was confirmed as IgM using ELISA (not shown). Another mouse harbored a faint M-spike (lower panel, lane 4).

**Supplemental Figure 5.** Flow-cytometric evaluation of surface immunoglobulin expression on malignant CD138^+^ cells harvested from 2 tumor-harboring BCL2^+^IL6^+^AID^-^ mice. Spleen and axillary, cervical and inguinal lymph node cells were obtained from two lymphoma-bearing mice, gated on forward scatter (FSC) and side scatter (SSC) followed by exclusion of doublets based on FSC-A/W analysis (not shown). Cells were identified as CD138^+^B220^+^ plasmablasts or CD138^+^B220^-^ plasma cells and labeled with antibodies to IgM, IgG and IgA. The abundance of plasmablasts and plasma cells ranged from 1.5% to 3.75% in the first mouse and 1.72% to 4.2% in the second mouse. The flow result demonstrates that the great majority of these cells (range 93.2% to 99.7%) expressed surface IgM. Expression of IgG or IgA was not detected (not shown).

**Supplemental Figure 6.** Immunohistopathology of tumor cell infiltrates in the liver of a lymphoma-harboring BCL2^+^IL6^+^AID^-^ mouse.

**(a)** Representative low-power immunofluorescence images (2X) of an OCT-embedded cryosection of a liver specimen labeled with a FITC-conjugated antibody to IgM. IgM^+^ cells aggregate in large clusters in portal tracts. One cell cluster is indicated by red rectangle.

**(b)** High-power microphotographs (40X) of serial FFPE sections from another liver specimen of the same mouse. The lumen of the portal vein and an area with normal liver parenchyma are located on the left and right margin of the 4 images shown, respectively. Situated in between are Ki67^+^ and/or CD138^+^ tumor cells (labeled brown) as well as Mac2^+^ (macrophages) and CD3^+^ (T lymphocytes) stroma cells.

**Supplemental Figure 7.** Two cases of splenic histiocytic sarcoma (HS) in BCL2^+^IL6^+^AID^-^ mice. Presented are H&E-stained FFPE tissue sections of mice with co-existing lymphoma and HS. In Case 1 to the left, lymphoma (top) and HS (bottom) are separated by a thick red line in the 4X image. The center image (40X) presents a portion of the HS at higher magnification. The sarcoma contains 3 multinucleated tumor giant cells, a hallmark of HS. The cells are encircled and shown at 200X at the bottom. In Case 2, the HS area is indicated by a yellow line in the top panel. The 40X image contains a piece of a B cell follicle and sarcoma to the left and right, respectively. Three multinucleated tumor giant cells (indicated by colored circles) are demonstrated at high power at the bottom. Evidence for a common clonal origin of B-lymphoma and HS in humans has been repeatedly reported (e.g., in patients with follicular lymphoma, as shown by Feldman *et al*., Blood 2008, 111:5433-5439), but whether this is also the case in the mice presented here is not known.

**Supplemental Figure 8.** Integrated FDG-PET and CT imaging of lymphoma-harboring BCL2^+^IL6^+^AID^-^ mice.

**(a)** Maximal projection PET/CT images (MIP) and three dimensional renderings of image stacks (3D) of two tumor-bearing mice (left and center panels) next to a normal mouse that was used as control (right panel). Tumor-bearing mouse 1 contains metabolically active (FDG^+^) peripheral lymph nodes (indicated by red arrowheads) that form a large confluent package in the cervical region. In contrast, tumor-bearing mouse 2 harbors grossly enlarged spleen and abdominal lymph nodes but exhibits little evidence for peripheral lymph node involvement. For imaging, the mice were anesthetized, administered ^18^F-FDG via the lateral tail vein and placed prone in a heated chamber in the scanner’s gantry. PET list mode data were acquired for 15 min using an Inveon small-animal PET/CT/SPECT imaging system (Preclinical Solutions, Siemens Healthcare Molecular Imaging, Knoxville, TN). In the same workflow, a CT image was acquired for attenuation correction purposes. Images were reconstructed using a 3D OP-MAP algorithm. Images were analyzed using PMOD v3.2 software.

**(b)** Determination of PET image parameters of the 2 tumor-bearing mice from panel a indicated peak total lesion glycolysis (TLG) in spleen. Maximal standardized FDG uptake (SUVmax), average standardized FDG uptake (SUVave) and metabolic tumor volume were measured for 3 tissues: cervical lymph nodes (CLN), spleen (SPL) and mesenteric lymph node (MLN). TLG was calculated as product of SUVmax and metabolic tumor volume.

**(c)** Necropsy photographs of 5 representative tumor-bearing BCL2^+^IL6^+^AID^-^ mice sacrificed at 3 different stages of tumor progression in terms of peripheral lymph node (LN) involvement. However, all 3 stages have a big abdominal tumor load, caused in large measure by spleen and mesenteric LN enlargement, in common. The most advanced progression stage, exemplified by cases 1113 and 1114, is generalized LN enlargement including nodes in the upper body half (cervical, axillary, brachial) and lower body half (inguinal, popliteal). A middle stage, represented by cases 1059 and 1062, affects the upper but not lower body half. Note the massive enlargement of cervical nodes in contrast to little if any involvement of inguinal nodes. The least advanced stage, exemplified by case 1144, exhibits little if any involvement of peripheral nodes.

**Supplemental Figure 9.** Genetic pathway analysis of the TME using Ingenuity Pathway Analysis (IPA) as research tool.

**(a)** Bar diagram of top-26 canonical pathways.

**(b)** NF-κB complex network diagram revealed by IPA’s network analysis module. Genes up-regulated are in green. Intensity of color is descriptive of the level of up-regulation. Straight and dashed lines indicate direct and indirect interactions, respectively.
