# Supplementary material for: Transgenic mouse model of IgM+ lymphoproliferative disease mimicking Waldenström macroglobulinemia
Source: Blood Cancer J. 2016 Nov 4;6(11):e488–. doi: 10.1038/bcj.2016.95 (PMC5148059; doi:10.1038/bcj.2016.95)
Supplement: Supplementary Table 1 [file bcj201695x2.docx]

**Supplemental Table 1:** Complete Blood Count (CBC) hematological survey values of tumor-bearing BCL2^+^IL6^+^AID^-^ transgenic (TG) mice (n=12) compared to normal (male/female) inbred BALB/cByJ mice included in The Jackson Laboratory Mouse Phenome Database (phenome.jax.org). Blood cell and differential cell counts were determined using the Bayer ADVIA 120 hematology analyzer system.

| **Category** | **Zz** | **Unit** | **TG Mice**  **Mean ± SD** | **Mean of Controls**  **Male/Female** | **Ratio**  **TG vs. Control ^1^** |
| --- | --- | --- | --- | --- | --- |
| WBC | White Blood Cells | 10^3^ / μl | 21.0 ± 4.26 | 3.52 / 2.79 | 6.65 |
| RBC | Red Blood Cells | 10^6^ / μl | 7.25 ± 1.58 | 10.5 / 10.8 | 0.681 |
| HGB | Hemoglobulin | g / dL | 10.9 ± 2.30 | 16.8 / 17.0 | 0.645 |
| HCT | Hematocrit | % | 34.2 ± 7.51 | 48.2 / 48.5 | 0.707 |
| MCV | Mean Corpuscular Volume | fL | 47.2 ± 0.67 | 45.9 / 45.0 | 1.04 |
| MCH | Mean Corpuscular Hemoglobin | pg | 15.0 ± 0.20 | 16.2 / 15.9 | 0.935 |
| MCHC | MCH Concentration | g / dL | 31.8 ± 0.25 | 35.9 / 35.4 | 0.892 |
| PLT | Platelets | 10^3^ / μl | 541 ± 23.3 | 956 / 1041 | 0.542 |
| MPV | Mean Platelet Volume | fL | 8.10 ± 0.36 | 6.49 / 6.91 | 1.21 |
| NEUT | Neutrophils | 10^3^ / μl | 2.23 ± 1.00 | 0.768 / 0.668 | 3.11 |
| LYMPH | Lymphocytes | 10^3^ / μl | 13.6 ± 4.35 | 2.58 / 1.98 | 5.96 |
| MONO | Monocytes | 10^3^ / μl | 2.74 ± 0.21 | ~0.0394 | 69.5 |
| EO | Eosinophils | 10^3^ / μl | 0.18 ± 0.05 | ~0.0892 | 2.02 |
| BASO | Basophils | 10^3^ / μl | 0.15 ± 0.04 | ~0.00878 | 17.1 |
| RET | Reticulocytes | % | 5.92 ± 1.91 | 3.05 / 2.92 | 1.98 |

^1^ The TG-to-Control ratios (rightmost column) are based on the mean of both genders (M/F).
