# Supplementary material for: Transgenic mouse model of IgM+ lymphoproliferative disease mimicking Waldenström macroglobulinemia
Source: Blood Cancer J. 2016 Nov 4;6(11):e488–. doi: 10.1038/bcj.2016.95 (PMC5148059; doi:10.1038/bcj.2016.95)
Supplement: Supplementary Table 2 [file bcj201695x3.docx]

**Supplemental Table 2:** Serum levels of cyto- and chemokines (pg/ml) in tumor-bearing BCL2^+^IL6^+^AID^-^ mice (n = 12) compared to normal C mice used as controls (n = 4). Mean values were compared using *t* test according to Mann-Whitney. Correlation of cytochemokine values with survival of BCL2^+^IL6^+^AID^-^ mice were determined by Pearson’s linear correlation analysis; *p* values smaller than 0.05 are indicated, with corresponding correlation coefficients shown in the last column.

| **Cyto- or chemokine**  **symbol** | **Mean ± SD**  **BCL2^+^IL6^+^AID^-^ mice** | **Mean ± SD**  **controls** | **Mann-Whitney *p*** | **Survival correlation (Pearson)** | **Survival fit**  **R^2^** |
| --- | --- | --- | --- | --- | --- |
| βFGF | 14.3 ± 39.2 | 4.60 ± 4.64 | n.s. | 0.0007 | 0.741 |
| CD27 | 2047 ± 6577 | 15.9 ± 25.0 | n.s. | 0.0008 | 0.734 |
| CD27L | 1366 ± 3620 | 121 ± 151 | n.s. | 0.0005 | 0.753 |
| CD40 | 145 ± 287 | 0.175 ± 0.350 | 0.005 | 0.0008 | 0.732 |
| CD40L | 32.1 ± 23.6 | 23.0 ± 3.89 | n.s. | n.s. | - |
| EGF | 63.9 ± 138 | 33.1 ± 65.5 | n.s. | 0.0003 | 0.783 |
| GCSF | 12.8 ± 35.5 | 2.88 ± 1.72 | n.s. | 0.0059 | 0.588 |
| IL-10 | 19.1 ± 14.0 | 22.3 ± 11.6 | n.s. | 0.0014 | 0.697 |
| IL-12 p70 | 1.51 ± 4.00 | 0.750 ± 1.43 | n.s. | 0.0016 | 0.686 |
| IL-17 | 1.15 ± 1.95 | 9.85 ± 16.2 | n.s. | 0.0348 | 0.407 |
| IL-1Ra | 4.53 ± 4.22 | 0.450 ± 0.252 | 0.007 | n.s. | - |
| IL-2 | 10.3 ± 21.8 | 3.15 ± 3.76 | n.s. | <10^-4^ | 0.851 |
| IL-2Ra | 465 ± 174 | 170 ± 41.5 | 0.013 | n.s. | - |
| IL-5 | 14.2 ± 9.31 | 5.28 ± 3.99 | n.s. | n.s. | - |
| IL-6 | 1.73 ± 1.07 | 1.45 ± 0.480 | n.s. | n.s. | - |
| MIP-1a | 1832 ± 5598 | 44.9 ± 82.8 | n.s. | <10^-4^ | 0.892 |
| RANTES | 120 ± 128 | 22.9 ± 11.4 | 0.008 | n.s. | - |
| SDF-1a | 646 ± 716 | 591 ± 488 | n.s. | 0.0008 | 0.734 |
| TRANCE | 13.3 ± 13.6 | 24.8 ± 24.2 | n.s. | n.s. | - |
| VEGF | 95.0 ± 35.4 | 196 ± 48.4 | 0.002 | n.s. | - |

List of abbreviations:

CCL, chemokine (C-C motif) ligand

IL, interleukin

IFN, interferon

MCP, monocyte chemotactic protein

TNF, tumor necrosis factor
