# Supplementary material for: Transgenic mouse model of IgM+ lymphoproliferative disease mimicking Waldenström macroglobulinemia
Source: Blood Cancer J. 2016 Nov 4;6(11):e488–. doi: 10.1038/bcj.2016.95 (PMC5148059; doi:10.1038/bcj.2016.95)
Supplement: Supplementary Figures [file bcj201695x4.pdf]

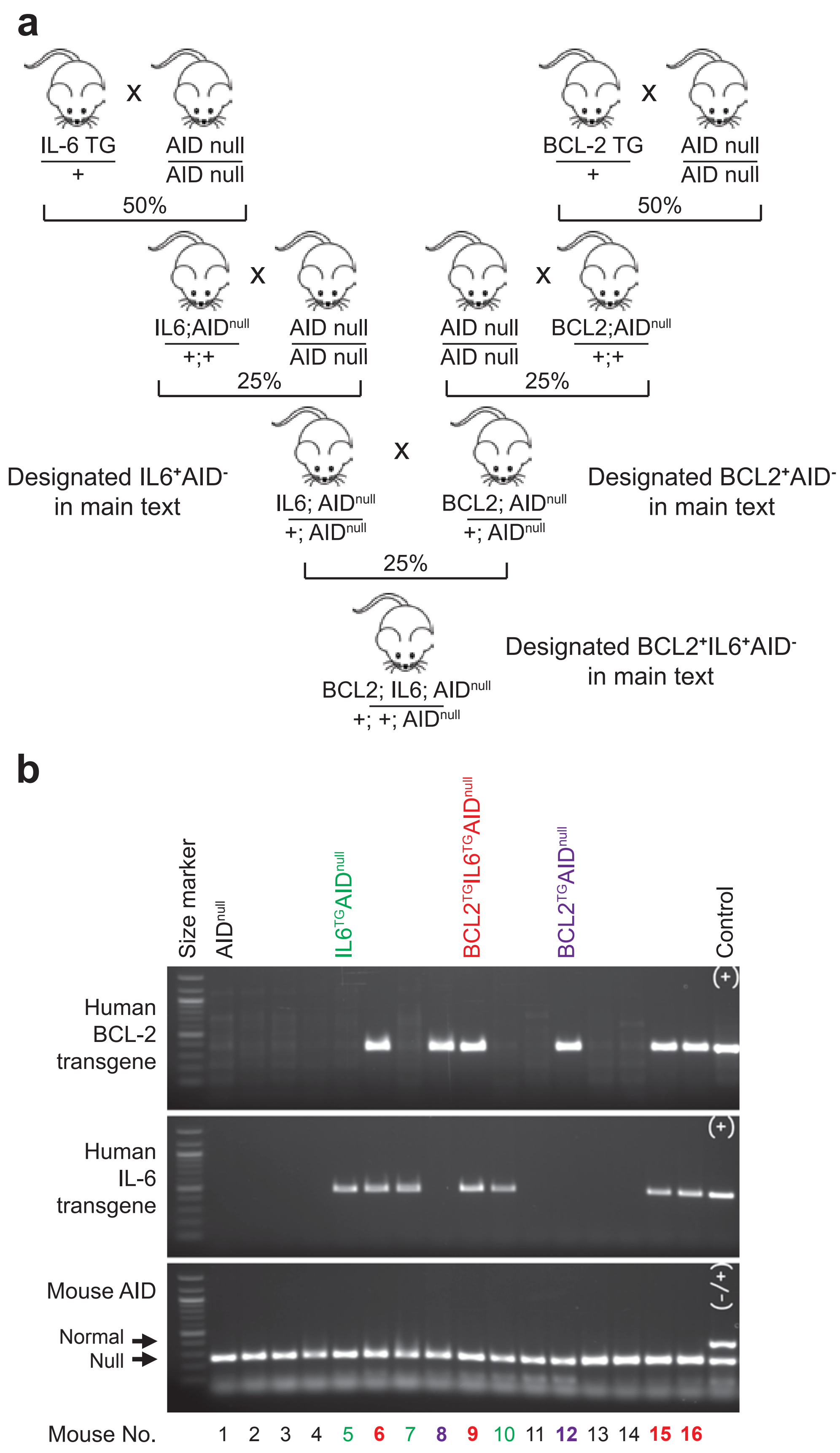

Supplemental Figure 1: Tompkins *et al.*

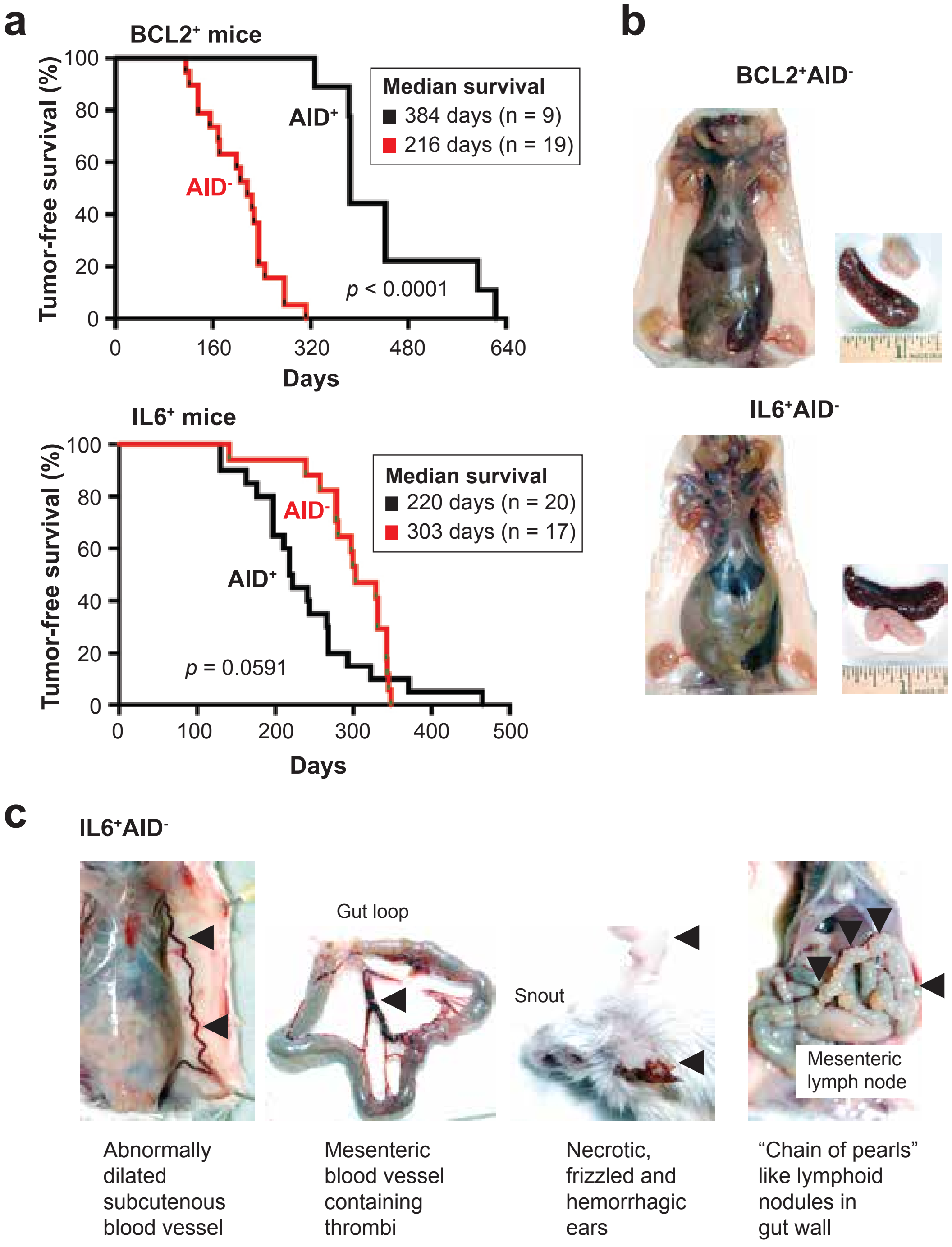

Supplemental Figure 2: Tompkins ***et al.***

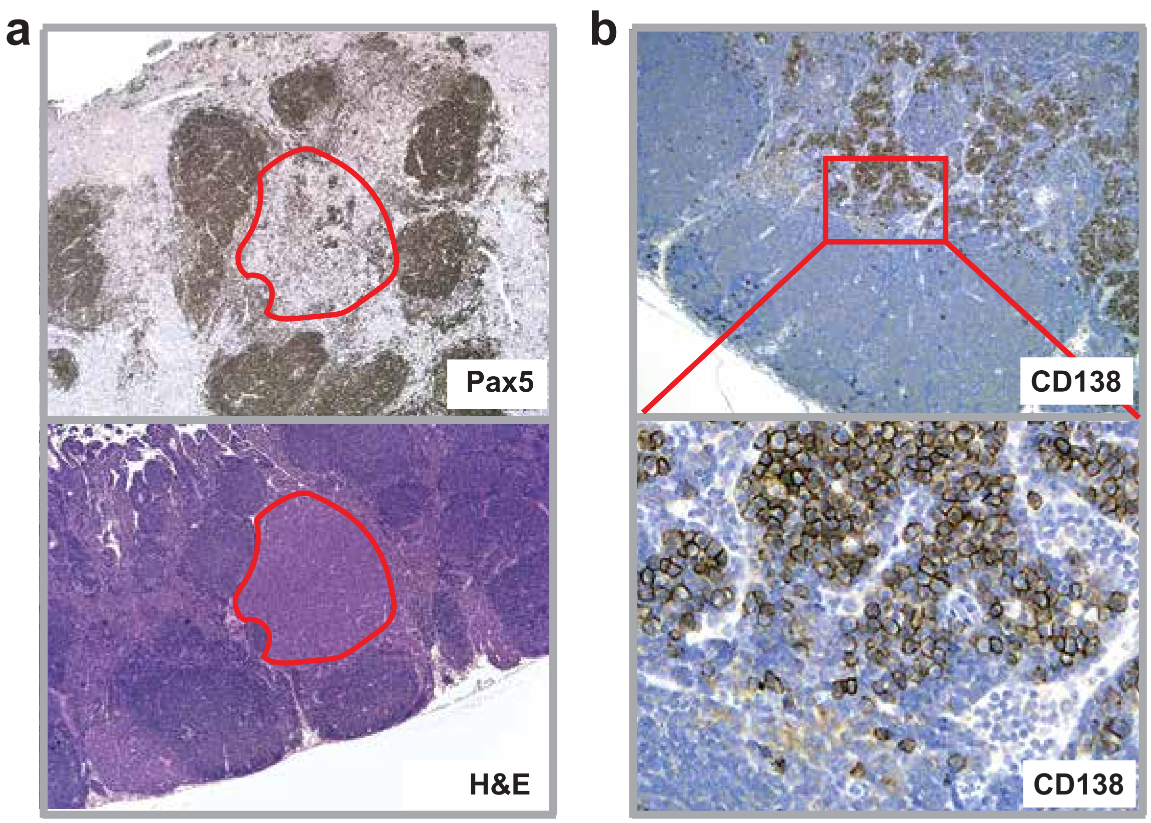

Supplemental Figure 3: Tompkins *et al.*

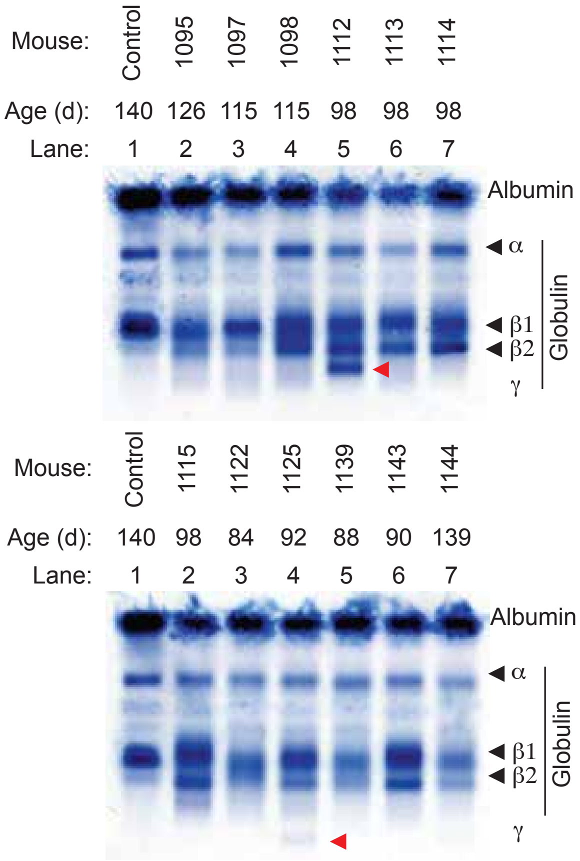

Supplemental Figure 4: Tompkins *et al.*

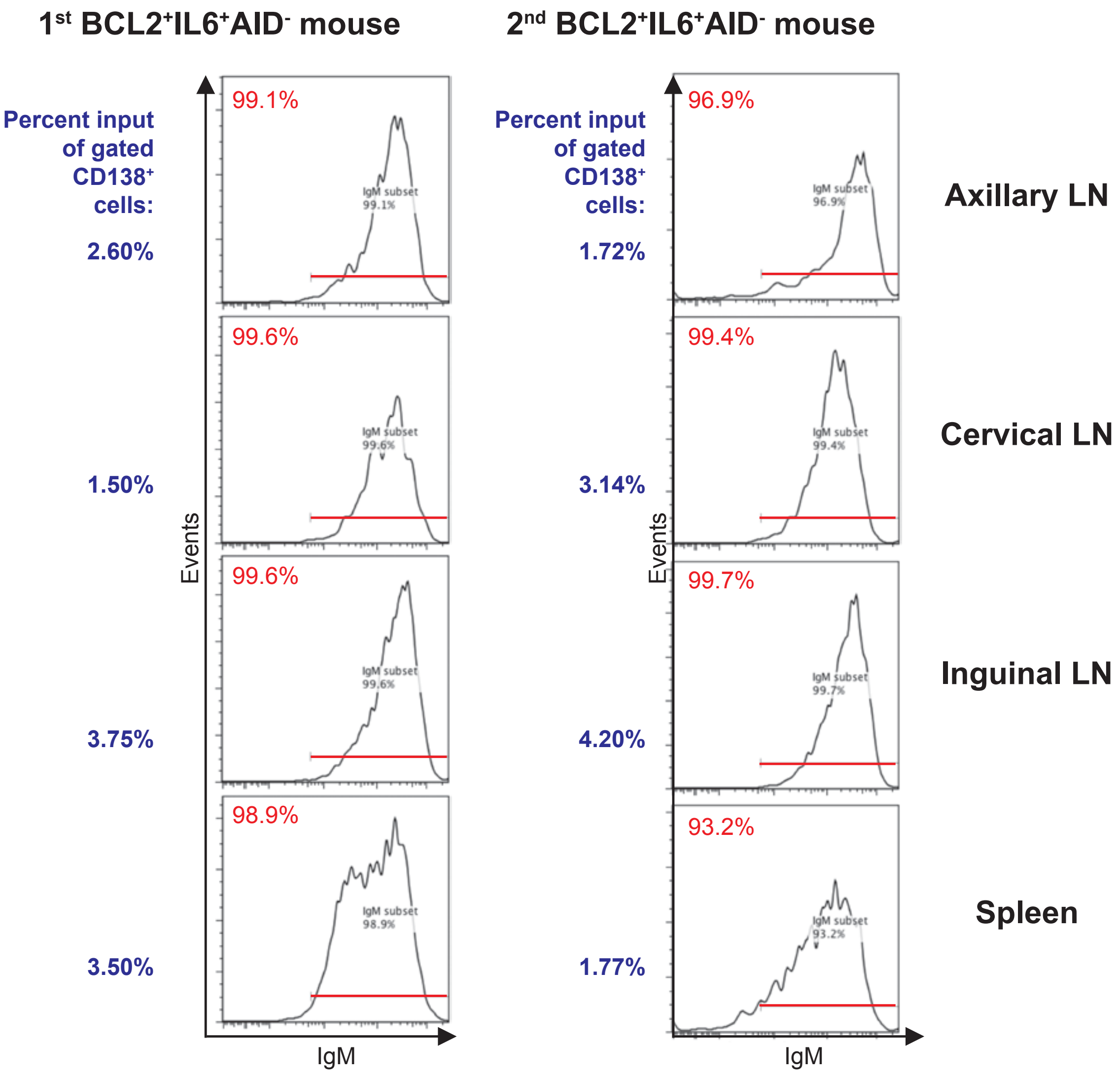

Supplemental Figure 5: Tompkins *et al.*

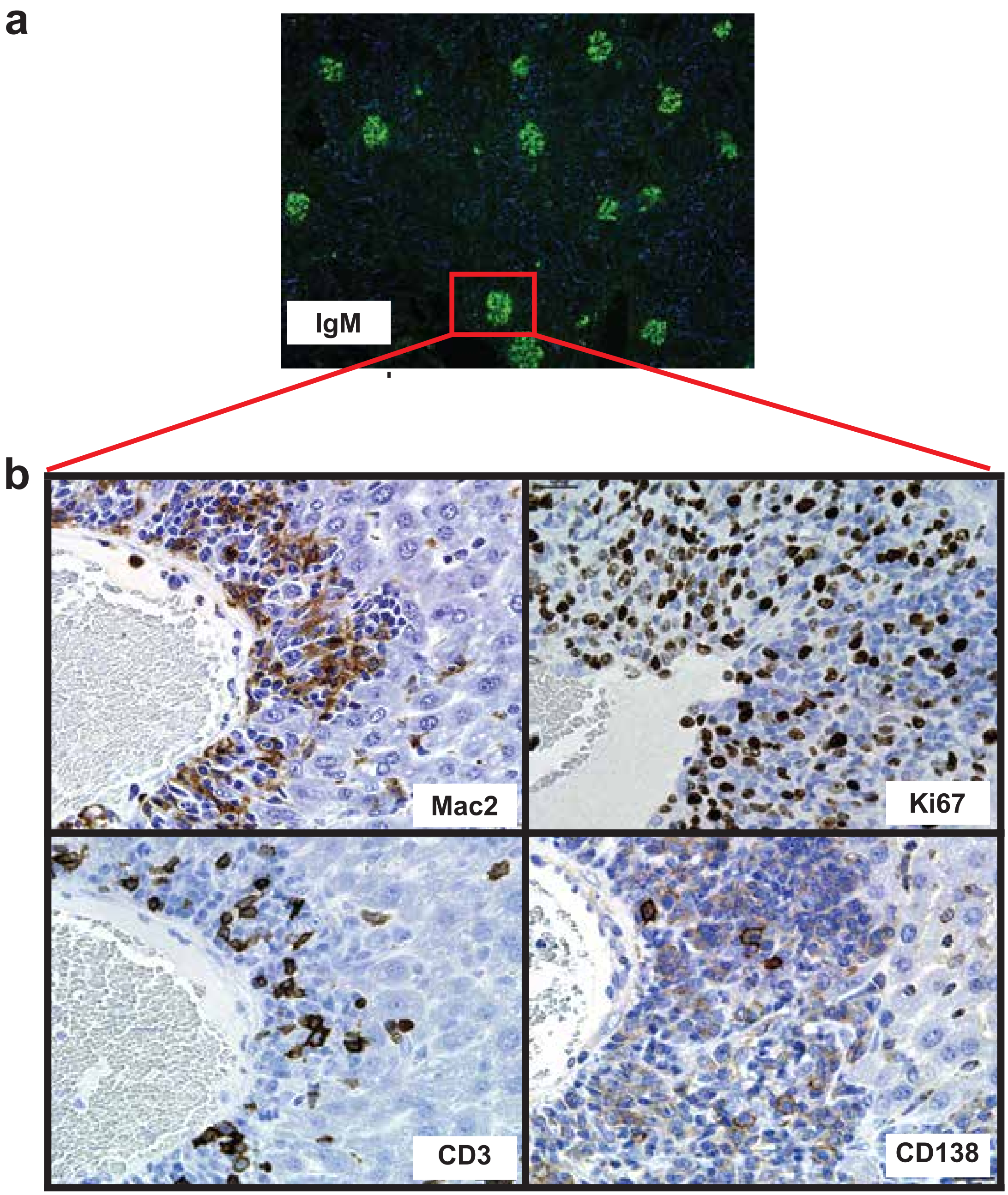

Supplemental Figure 6: Tompkins *et al.*

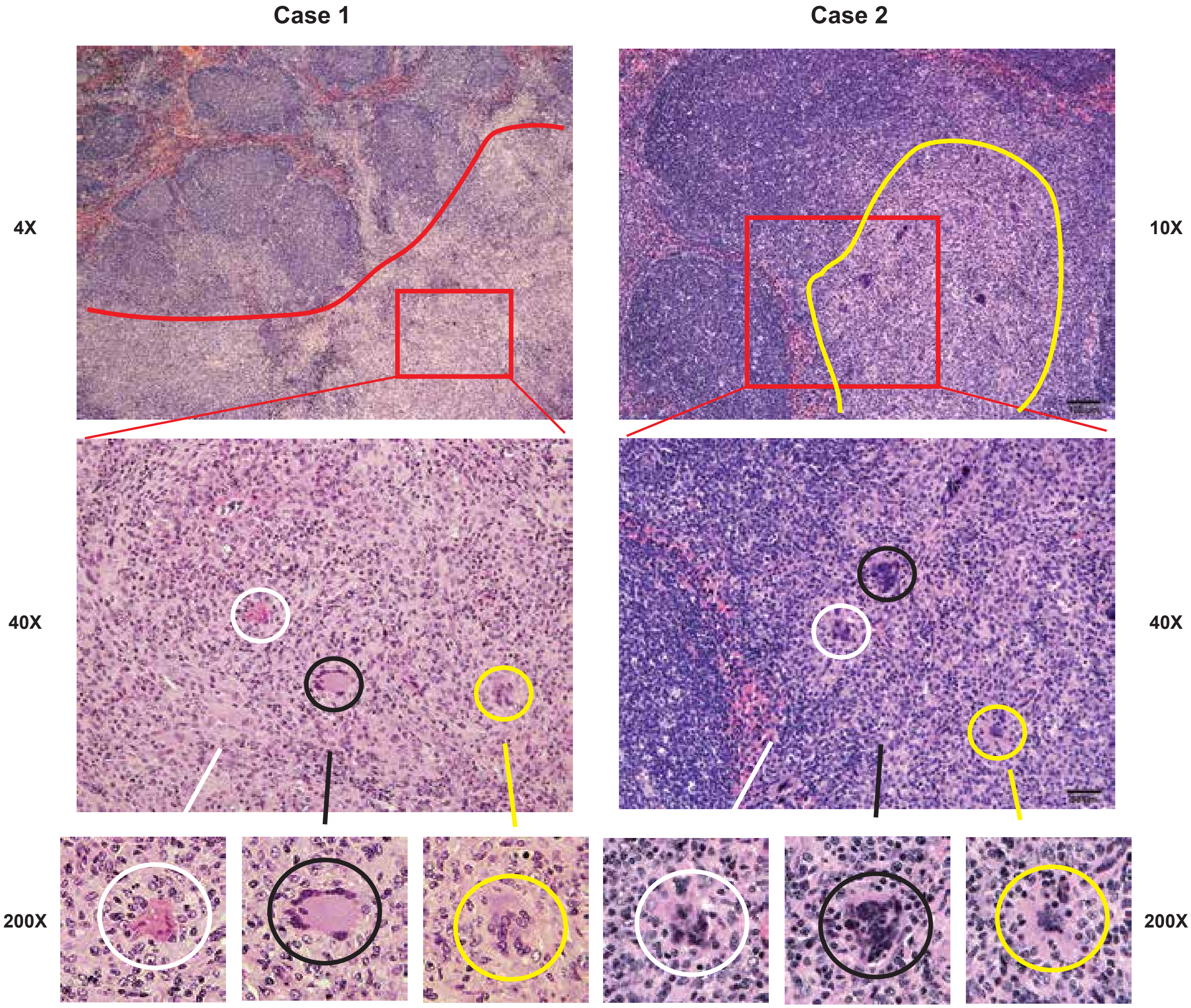

Supplemental Figure 7: Tompkins *et al.*

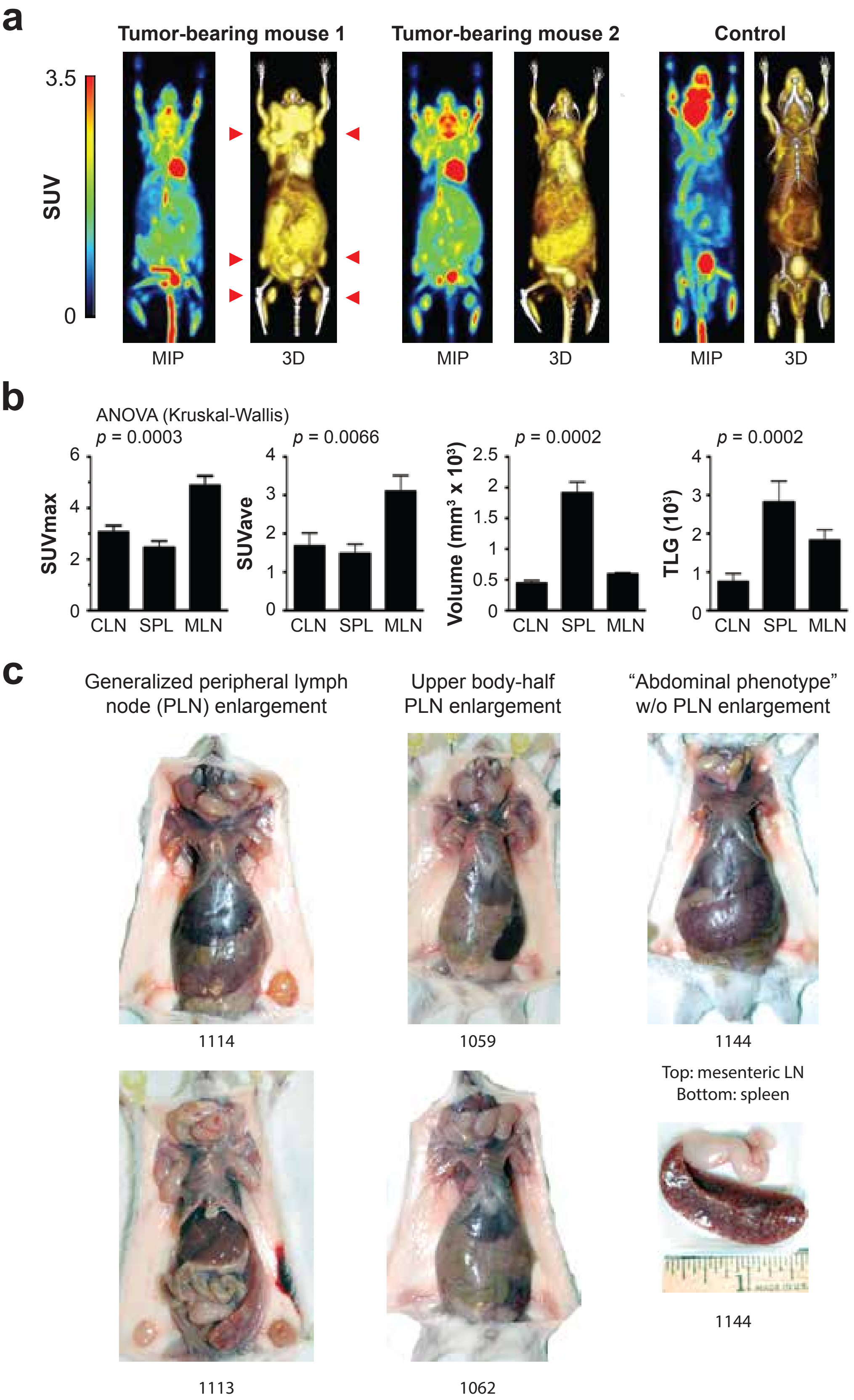

Supplemental Figure 8: Tompkins *et al.*

a

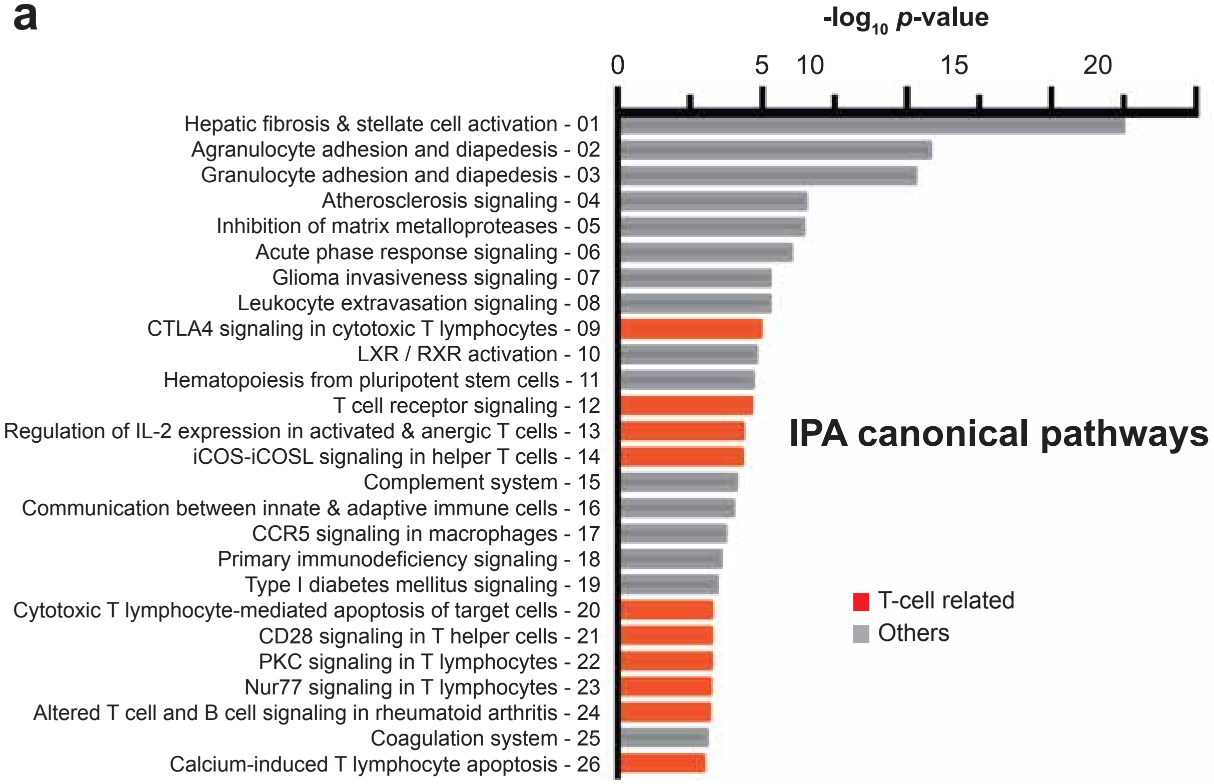

b

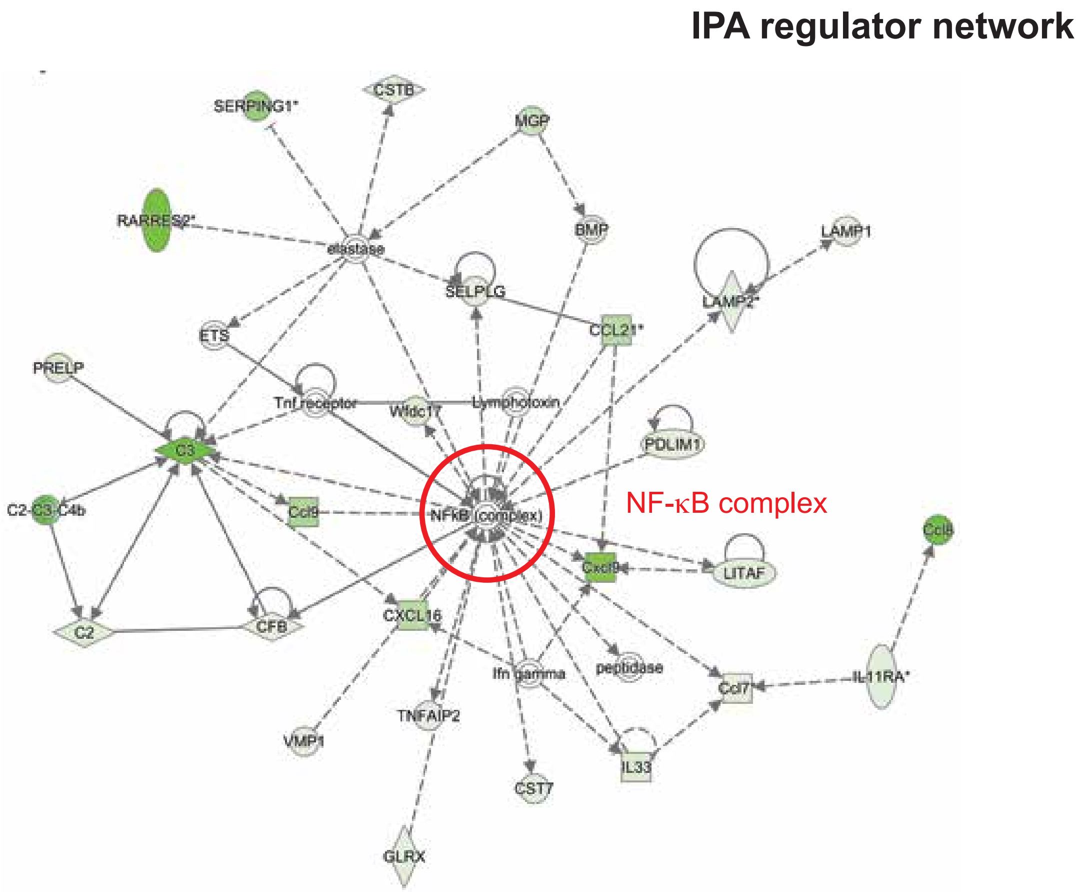

Supplemental Figure 9: Tompkins *et al.*
